# A promising microbial α-amylase production, and purification from *Bacillus cereus* and its assessment as antibiofilm agent against *Pseudomonas aeruginosa* pathogen

**DOI:** 10.1186/s12934-023-02139-6

**Published:** 2023-08-01

**Authors:** Amal M. Abo-Kamer, Ibrahim S. Abd-El-salam, Faten A. Mostafa, Abd-El-Rahman A. Mustafa, Lamiaa A. Al-Madboly

**Affiliations:** 1 Department of Pharmaceutical Microbiology, Faculty of Pharmacy, Tanta University, Tanta, Egypt; 2 Departemet of Chemistry of Natural and Microbial Products, National Research Centre, Dokki, Cairo, Egypt

**Keywords:** Amylase, Soil bacteria, Partial purification, Amylolytic activity, RSM, Antibiofilm, CLSM

## Abstract

**Background and aim:**

The purpose of the current study is to isolate a heavily amylase-producing bacteria of the genus *Bacillus* from soil samples, optimize the production of the enzyme, purify it, and evaluate its activity against biofilm-producing bacteria. A total of 12 soil samples were collected and screened for promising *Bacillus* species with good amylolytic activity. Isolation was done by serial dilution and plating technique and amylolytic activity was determined by starch agar plate method. Among the 12 *Bacillus* isolates recovered from soil samples, 7 showed positive α-amylase production. The best isolate that recorded the greatest amylolytic activity was selected for further studies. This isolate was identified by 16S rRNA sequencing as *Bacillus cereus* and registered under gene bank accession number OP811897. Furthermore, the α-amylase enzyme was produced by a submerged fermentation technique using best production media and partially purified by ammonium sulfate and chilled ethanol and molecular weight had been determined by SDS-PAGE gel electrophoresis. The production of α-amylase was optimized experimentally by one-factor at a time protocol and statistically by Plackett–Burman design as well as RSM CCD design. Data obtained from OFAT and CCD revealed that α-amylase activities were 1.5- and twofold respectively higher as compared to un-optimized conditions. The most significant factors had been identified and optimized by CCD design.

**Results:**

Among the eleven independent variables tested by PBD, glucose, peptone, (NH4)_2_SO4, and Mg SO_4_ were the most significant parameters for α-amylase production with an actual yield of 250U/ml. The best physical parameters affecting the enzyme production were incubation time at 35 °C, and pH 5.5 for 48 h. The partially purified enzyme with 60% ammonium sulphate saturation with 1.38- fold purification showed good stability characteristics at a storage temperature of 4 °C and pH up to 8.5 for 21 days. Antibiofilm activity of purified α-amylase was determined against *Pseudomonas aeruginosa* (ATCC 35659) by spectrophotometric analysis and CLSM microscopic analysis. Results demonstrated biofilm inhibition by 84% of the formed *Pseudomonas* biofilm using a microtiter plate assay and thickness inhibition activity by 83% with live/Dead cells percentage of 17%/83% using CLSM protocol.

**Conclusions:**

A highly stable purified α-amylase from *B. cereus* showed promising antibiofilm activity against one of the clinically important biofilm-forming MDR organisms that could be used as a cost-effective tool in pharmaceutical industries.

## Introduction

Microbial enzymes have grown in popularity due to the simplicity of being isolated in large quantities, inexpensive production in a short amount of time, and stability under various complicated circumstances. Furthermore, their compounds are more manageable and less hazardous. Biosynthesized by microorganisms, amylases exhibit unique properties including thermophilic, thermotolerant, alkaline, and acidophilic properties. The primary benefits of using microorganisms to produce amylases are the cost-effective bulk production procedures and the ease of manipulating microbes to gain enzymes with the desired properties. The extensive availability of amylase substrates from inexpensive plant sources makes the dormant applications of the enzyme more cost-effective. So that, to gain optimal yield of an enzyme, it is necessary to develop an appropriate medium and culture conditions. Enzyme production and bacterial proliferation require starch or monosaccharide sugars as a carbon source and ammonium salts either organic or inorganic compounds as a nitrogen source [1].

The optimization of the various parameters and manipulation of the medium are two of the most important methods for increasing the production of α-amylase in massive quantities. The generation of α-amylase is known to be influenced by a number of distinct physical and chemical variables, such as the pH level, temperature, incubation time, and carbon and nitrogen supplies. Numerous researchers have researched the generation of α-amylase using the submerged fermentation approach, and a variety of physiochemical parameters play a role in this process. It is vital to improve the system's effectiveness and improve output without elevating production expenses in order to meet the industry's rising demand. Bacteria produce α-amylase more cheaply and effectively than other microorganisms within the wide range of microorganisms that do so. Statistical design experiments and mathematical techniques like Plackett–Burman and CCD designs are widely used in the discipline of microbial biotechnology. The goal of the response surface methodology (RSM), a technique used to model problems, is to optimize responses that are influenced by various variables. The main steps include carrying out statistically planned experiments, expecting experimentally identified response data into a quadratic framework, assessing response, and evaluating model significance. Because RSM uses fewer experimental trials for the prediction and evaluation of coupled interactions between variables, it makes the optimization process much easier for industrial applications. Response surface contour graphs have frequently been used to maximize the production of microbial enzymes because they make parameter interaction evident [2].

Microbial biofilms have been an inevitable and essential hazard to human beings, environmental, and natural health. This is because it has been linked to numerous infectious diseases. In addition, they are infectious and can cause nosocomial infections. Biofilms also exhibit a high level of antibiotic resistance. Enzymes are extremely biodegradable and innocuous to the environment, making them an effective method for biofilm removal. Amylase is recognized as a digestive enzyme that hydrolyzes the glycosidic bonds of starch to form maltotriose glucose, dextrin, and maltose; therefore, it is classified as a glycosidic hydrolase. It is more advantageous for microbes to produce α-amylase due to their high production rate and their ability to be readily engineered into desired products. Compared to aquatic environments, the majority of microorganisms are found in the soil, which is the primary component of the terrestrial environment. This is due to its increased organic and inorganic material content. *Bacillus* sp. is an excellent choice as a source for α-amylase producers such as *B. subtilis*, *B. cereus*, and *B. amyloliquefaciens*. Additionally, α-amylase obtained from *Bacillus* is thermostable, resistant to extreme pH, osmolality, and pressure, which is essential for industrial production [3].

The main goal of this study was to isolate a promising *Bacillus* species from extremely environmental circumstances and use it to produce α-amylase by a small-scale submerged fermentation in order to overcome these challenges and meet the demand. Additionally, the purification and the application of the test enzyme as an antibiofilm will be investigated.

## Materials and methods

### Media for isolation and optimization

Nutrient agar (Oxoid, UK) was used for the isolation of *Bacillus* from the soil, and starch agar (Oxoid, UK) was utilized in the detection of the amylolytic activity of the recovered organisms.

Five different media composition were selected to determine best basal media for α-amylase production [36].

*Media No.1 (M1)* composition (g/l).

Glucose, 20; yeast extract, 10; MgSO4.7H2O, 1; KH2PO4, 2.

*Media No.2 (M2)* composition (g/l).

Peptone, 10; starch, 10; yeast extract, 20; CaCl2, 0.05; MgSO4, 0.25; KH2PO4, 0.25; (NH4)_2_SO4, 0.25; FeSO4, 0.01 (control media).

*Media No.3 (M3)* composition (g/l).

Soluble starch, 10; peptone, 6; MgSO4, 0.5; potassium chloride, 0.5.

*Media No.4 (M4)* composition (g/l).

Soluble starch, 20; yeast extract, 5; magnesium sulphate, 1; CaCl2.2H2O, 0.2; NaCl, 1.

*Media No.5 (M5****)*** composition (g/l).

Soluble starch, 10; peptone, 25; KH2PO4.3H2O, 1.5; NaSO4, 1.5; 0.15; FeSO4.7H2O, 0.03; MnCl2.4H_2_O, 0.15; CaCl2.2H_2_O, 0.45.

### Collecting samples and isolating bacteria

Twelve soil samples were assembled from 10th of Ramdan city, 3rd industrial zone, Sharqiya governate, Egypt. They were obtained from different depths, 0.5 g of every specimen has been suspended in 9 ml of sterile distilled water. The later suspension was subjected to heat shock for 1 h at 60 °C in order to kill all non- spore forming bacteria. Then, 0.5 ml of each test suspension was transferred and cultivated on a nutrient agar (NA) plate, spread uniformly by a lab glass stick and incubated at 37 °C for 24 h to ensure sufficient bacterial growth. To effectively isolate the desired bacterial species, soil solutions were diluted by performing serial dilutions and streak plating, pure colonies of target bacterial cells were obtained. Samples were then reinoculated on NA plates and incubated at 37 °C for 24 h. Next after incubation, colonies were sub-cultured on NA plates and stored at 4 °C for future use [4, 5].

### Primary qualitative assessment and selection of lhe potent α-amylase producer

Isolates were screened for α-amylase producing ability by inoculation of bacterial colonies on primary starch agar media with composition (g/l): soluble starch,10; peptone, 5; beef extract, 5; NaCl, 2; MgSO4, 2; agar, 10; with adjusting pH at 7 and incubated at 37 °C for 24 h. All working media were supplied from (Oxoid, UK). Immediately after incubation, plates were flooded with sterile 0.05 M lugol’s iodine solution (Gram's iodine: 5 gm potassium iodide powder was dissolved in 100 ml of sterile distilled water then 3.23 g of iodine crystals was added to the solution, mix well and complete the volume with distilled water, store at the ambient temperature). Based on the clear halo of hydrolysis around bacterial colonies, α-amylase positive isolates were determined and recorded. Out of the recovered *Bacillus* isolates, the best amylase-producing one was selected and sub-cultured on NA, then stored for further experimental studies [4, 5].

### Amylase colorimetric quantitative assay

According to Miller et al.'s illustration in 1959, the amount of reducing sugar released from soluble starch was measured to perform the amylase assay [6]. Bacterial colonies have been inoculated in previous culture media. After that, incubation has been done for 24 h at 37 °C. Media was centrifuged at 5000 rpm for 10 min using (ZM200, Germany) centrifuge to obtain the supernatant. After that, reaction mixture samples were taken (1 ml of enzyme from supernatant + 1 ml of 1% soluble starch) and incubation was done at 35 °C in shaking water bath for 15 min. Then, to stop the reaction, 2 ml of freshly prepared dinitrosalicylic Acid (DNS) solution was added. Tubes were placed back in a water bath at 100 °C for 15 min in order to develop the color and allowed to cool at room temp for 20 min and OD reading were gathered. The supernatant was then collected to be used as a crude enzyme for the upcoming procedures. OD has been determined at 540 nm utilizing a spectrophotometer (Jenway, 6305-Japan). By plotting absorbance at 540 nm against the amounts of maltose released (g), a maltose standard curve was created to determine the concentration of maltose formed in each solution. Amylase activity was then calculated and so one unit of enzymatic activity is equivalent to the amount of the enzyme needed to catalyse the synthesis of reducing sugar under typical assay circumstances, which is equal to 1 µmol/min of maltose [4, 6].

The following equation was applied to allow for estimation of the enzyme activity; enzyme activity

$$ {{{\text{IU}}} \mathord{\left/ {\vphantom {{{\text{IU}}} {{\text{ML}}}}} \right. \kern-0pt} {{\text{ML}}}}{\text{ = Amount of reducing sugar}} \times {{{1000}} \mathord{\left/ {\vphantom {{{1000}} {{\text{Molecular weight of glucose}} \times }}} \right. \kern-0pt} {{\text{Molecular weight of glucose}} \times }}{\text{time}} $$

### Morphological, biochemical and genetic identification of α-amylase producing isolate

To get information regarding morphology, bacterial colonies have been stained with the standard Gram-stain and investigated under an oil immersion high power light microscopy (100×magnification lens). Bacterial isolate was then characterized using standard biochemical tests (ex: (Indole, VP, citrate tests) for further identification [4, 5].

Using alkaline lysis protocol and heat shock technique, DNA was isolated from bacterial cultures [4]. Following that, 16S rRNA universal primers were utilized for amplification of the 16S rRNA gene sequence. 1 µL of template DNA and 1 µL of each primer were added and the samples have been loaded in Polymerase Chain Reaction (PCR) machine. The universal primers 5ʹ—ACGGGCGGTGTGTAC-3 as a forward primer and 5ʹ, -CAGCCGCGGTAATAC-3 as a reverse primer were used. Using (cycler 170–8740, USA), the fragmentary sequence of the 16S rRNA gene of isolate (1500 bps) was amplified. Thermal cycle was programmed as discussed: initial denaturation step at 94 °C for 6 min, annealing step at 56 °C for 30 s, extension step at 72 °C for 2 min and final extension step at 72 °C for 5 min. PCR was performed for 30 cycles and product was subjected to sanger sequencing using (ABI 3730 xl DNA sequencer, Germany) at GATC company. The 16S rRNA gene sequences have been aligned and sequence similarity has been compared to known microorganisms in the Gene Bank database of the National Center for Biotechnology Information utilizing Basic Local Alignment Search Tool (BLAST) [4, 7, 8].

### Enzyme production by submerged fermentation

#### Inoculum preparation and preliminary selection of enzyme production media

A culture sample of 5 days old of the selected *Bacillus* isolate with promising amylolytic activity (vegetative inoculums) has been utilized to inoculate 50 ml of fermentation production media in 250 ml Erlenmeyer flask. Basal media was adjusted to pH 6.5 before autoclaving using a pH-meter (CL-40 M, Japan). Additionally, α-amylase activities have been assessed after 48 h of cultivation at 35 °C. The basal production media (control) composition was in (g/l): soluble starch, 10; yeast extract, 20; CaCl_2_, 0.05; MgSO_4_, 0.25; peptone, 10; and KH_2_PO_4_, 0.25. Flasks were then incubated in rotary shaker fermenter at 35 °C for 24 h and 150 rpm in rotary shaker incubator (Stuart S1-500-UK). After that, in a refrigerated centrifuge (Hitachi CF16 RII-Japan), the suspensions of bacteria have been centrifuged at 10,000 rpm for 10 min. The supernatant has been subsequently collected and stored at 4ºC for further research as a crude extract for enzyme activity assay [9, 10].

Primary production factors which probably affects α-amylase production, ex: temperature, period of incubation, pH, sources of carbon and nitrogen, metal ions have been analyzed separately before optimization experiments, enzyme assay was performed according to standard assay procedures.

#### Optimization of culture media conditions and medium components for best α-amylase production by using (OFAT)

#### Effect of various fermentation media on α-amylase production

Production of α-amylase was performed by submerged fermentation process utilizing 5 fermentation media (Oxoid, UK) to determine basal control best media for running one factor at a time protocol experiments [11, 12].

#### Effect of various time periods on α-amylase production

Fermentation media for production of α-amylase were incubated at different times (24, 48, 72, 96, 120 h), enzyme samples (5 ml) were collected. Following that, determination of enzymatic activity was performed under standard ideal assay conditions. [11, 12].

#### Effect of various pH on α-amylase production

Regarding highest α-amylase activity, best media were incubated at different pH (4.5, 5.5, 6.5, 7.5, 8.5). samples were collected and enzyme activity was assessed. Considering all previous experiments, the supernatant has been extracted by centrifuging and utilized for measurement of the α-amylase activity under standard assay conditions. The un-inoculated flasks served as controls [13, 14].

#### Effect of various temperatures on α-amylase production

Fermentation media of interest were incubated at different temperature degrees: (20, 25, 30, 35, 40, 45). Samples were collected and enzymatic activity has been assessed under standard assay conditions. other factors were constant at their optimal values [13, 14].

#### Effect of various carbon sources on α-amylase production

Bacterial cells were grown on basal media (M2) that contained various carbon sources, such as glucose, sucrose, and maltose, in order to produce α-amylase. Fermentation media with different carbon sources at concentrations (5 g/L) were inoculated and then incubated at 35 °C for 2 days with agitation speed of 150 rpm. Extracted culture filtrate was then used for assay of extracellular α-amylase quantitatively. Other factors remained constant at their optimal values [13, 15].

#### Effect of various nitrogen sources on α-amylase production

Different nitrogen sources either organic or inorganic (ex: peptone, yeast extract, beef extract, (NH4)_2_SO4 and KNO3 were supplied individually into fermentation broth media at concentrations (5 g/l), flasks having the fermentation media were incubated at 35 °C for 48 h with agitation speed equals to 150 rpm and enzyme activity was investigated. Other factors were adjusted at their optimal values [13, 15].

#### Effect of various metal ions on α-amylase production

Fermentation media have been inoculated with various metal ions: (BaCl2, CaCl2, MnCl2, ZnCl2, NiCl2, MgSO4, and CuSO4) at concentrations 10 mM. Samples were collected and enzymatic activity was evaluated under ideal assay conditions. Other variables remained constant at their optimal levels [16].

### Multi factorial experiments for optimization of α-amylase production

The optimization protocol was divided into three basic phases. The first phase was done in accordance with Placket and Burman (1964) for evaluation of the relative importance of several media components affecting α-amylase production. The second method involved selecting the most important PBD components for further calculation of their ideal levels using CCD and related contour maps. Lastly, utilizing the computational statistical analysis (ANOVA) to validate the model's fitness as measured by the determination coefficient (R2) [11, 17].

#### Plackett–Burman design (PBD)

This design looks at the impact of 11 various variables on the production of α-amylase, including starch, glucose, lactose, yeast extract, peptone, NH4SO4, CaCl2, KCl, MgSO4, CuSO4, and Tween 80. In a table where each row represents an experiment and each column indicates an independent variable, each of these variables was represented by two levels: a low level (− 1) and a high level (+ 1). All tests were carried out in 250 ml Erlynmeyer flasks with 50 ml of the fermentation medium, samples were gathered every 48 h at pH 5.5 and 35 °C, absorbance was measured at 540 nm by UV spectrophotometer, and enzyme activity was calculated using the previously discussed mathematical equation.

Plackett–Burman screening design is dependent on the equation of the first order polynomial model.

$$ Y = \, B_{o} + \, \Sigma B_{i} X_{i} $$

In this model, Y stands for the response to α-amylase activity, B0 for the model intercept, Bi for the linear coefficient, and Xi for the value of the relevant independent factor. Analysis of variance was used to evaluate the design's relevance. [18–20].

#### Optimizing α-amylase production using response surface methodology central composite design

By assessing the impacts of many parameters and their interactions, response surface methodology represents a widely acknowledged statistical model that may be used to optimize the experimental procedure. The Design Expert software (Version 7, Stat-Ease Inc., USA) was used to analyse and plot the response surface graphs. In order to establish RSM utilizing Central Composite Design (CCD), four variables—glucose, peptone, MgSO4 and (NH4)_2_SO4—were optimized to enhance production of α-amylase using the isolated *B. cereus* while other variables were held constant. The experimental design matrix was used to analyze the parameters on five levels: (0, + 1, − 1, + 2, − 2, for the central level, first high level, first low level, second high level, and second low level respectively). An overall of 30 trials were carried out to improve the process parameters. The results were assessed using the coefficient of determination (R2), the analysis of variance test, and contour response plots. The most well-liked second-order polynomial equation was created in order to match the experimental results and pinpoint the pertinent model terms.

$$ Y = \beta_{0} \Sigma \beta_{i} X_{i} + \Sigma \beta_{i} X_{i} \beta_{ij} + \Sigma X_{i} X_{j} $$

Provided that Y is the predicted response.β_0_, bi, and b_ij_ are model constant regression coefficients

X_i_ and X_j_ are the independent variables.

The experimental design facilitates the investigation of linear, quadratic, and cross-product effects of these parameters, as well as replication center points. Experiments were conducted using the variables under the model's expected circumstances in order to verify the model and results validity [21, 22].

### Partial purification of α-amylase enzyme

#### Ammonium sulphate precipitation and dialysis

The culture broth had been centrifuged at 10,000 rpm for 15 min at 4 °C, the clear supernatant has been pooled. After that, condensation under vacuum in a rotary evaporator was implemented for concentrating the enzyme protein. The undesirable proteins were then precipitated from the condensed broth using a water bath at 45 °C for 30 min. The resultant precipitate was then removed and the enzyme-containing supernatant was cooled. Using **(Dixon and Web’s approach 1964**), crude enzyme (supernatant) had been subjected to ammonium sulphate precipitation. Different saturation levels of (NH4)_2_SO4 (20, 40, 60, and 80%) in 0.01 M sodium phosphate buffer (pH 6.5) have been used for α-amylase precipitation. Respective amounts of (NH4)_2_SO4 were gradually added to the crude enzyme solution and placed in ice salt bath for 10 min with continuous stirring then these fractions were centrifuged at 10,000 rpm for 15 min at 4 °C to recover the precipitated protein, after centrifugation, the supernatant was discarded and the sediment fraction containing our protein was dissolved in 10 ml of 0.01 M Na3PO4 buffer (pH 6.5). The best fraction solution was subjected to membrane filtration by utilization of Millipore dialysis bags (Amicon company, Sigma Aldrich, USA) with 10,000 or 12,000 MW cut-off membranes after sealing and dialysis against phosphate buffer overnight with continuous stirring and periodic change of buffer till salt ions were removed. The dialyzed samples were filtered using a Millipore filter, and the fractions that were obtained were then redissolved in the same buffer and tested for enzyme activity using the usual DNS method. The protein content was then measured using Lowry method and BSA was used as a reference. Briefly, 1 ml of the test protein in buffer was added to 0.9 ml of reagent A, mixed and incubated at 50 °C for 10 min. After that, the mixture was cooled to room temperature then, 1 ml of reagent B was added for another 10 min. Following incubation at room temperature, 3 ml of reagent C were added for 10 min then, the absorbance of the mixture was measured at 650 nm. Next purification stages utilized the best fraction [23–25].

#### Precipitation by organic solvent using chilled ethanol

In this experiment, steps involved centrifugation and concentration as previously discussed were carried out and the supernatant with crude enzyme was precipitated using different proportions of chilled ethanol (0–25, 25–50, 50- 75 and 75–100).

The crude enzyme was taken in a glass beaker and precipitated by using different saturation levels of chilled at (− 20 °C) which was added slowly with continuous stirring at 4 °C for 15 min. Centrifugation was used to separate the precipitated protein fractions for 15 min at 4 degrees Celsius and 5000 rpm. The resulting pellets were used for amylase assay and also for protein quantification using Lowry technique where BSA was considered as a reference $\mathrm{9,25}.$

### Molecular weight determination of purified α-amylase enzyme

According to Laemmli [26], the purity and molecular weights of the amylases were evaluated employing SDS-PAGE with 7.5% (w/v) stacking gel and 10% (w/v) resolving gel. The 100 µL sample was mixed with 100 µL of sample buffer, boiled for 2 min, then chilled for 15 min before being injected into the device wells. Using a Bio-Rad mini protean tetra cell system with a 120 voltage, electrophoresis was performed. The run was stopped when the dye samples reached the bottom of the gel’s 0.3 to 0.5 cm limit, after which the gel was immersed in brilliant blue Coomassie dye (0.1% w/v Sigma Aldrich, Germany) and gently agitated for 2 h before being rinsed in a de-staining solution. The enzyme molecular weight had been calculated via utilizing low molecular weight calibrating kit (Bio-Rad) as protein markers (Catalogue number SDS 7B2, Sigma, Germany) that is made up of standard protein markers like myosin marker (205 kDa), phosphorylase marker (97 kDa), bovine serum albumin marker (66 kDa), ovalbumin marker (43 kDa), and carbonic anhydrase marker (29 kDa), trypsin inhibitor marker (20.1 kDa) and α-lactalbumin marker(14.2 kDa). By staining with Coomassie Brilliant Blue R-250 (0.2%), the separated protein bands have become visible [9].

### Enzyme characterization

#### Thermal stability

Thermal stability characters of this promising α-amylase had been done by enzyme incubation at temperatures values: (4, 35, 55, 65,75, 90 °C) for 2, 4 and 6 h. Residual activity was recorded every 15 min throughout this time. [8].

#### pH stability

pH activity of purified α-amylase has been tested by incubation of enzyme in different buffer solutions with different pH ranges (4.5–9.5) ex: Tris-buffer 0.1 M, acetone buffer 0.1 M for 2 h, 4 h, 6 h and residual activity was assessed at ideal conditions (pH 5.5, 35 °C) [8].

#### Storage stability

Storage stability of enzymes was tested by storing enzyme at 4ºC and 35 °C for 40 days and checking the residual activity every week [8].

### Antibiofilm activity of purified α-amylase

#### Determination of MBIC and MBEC of purified and commercial α- amylase enzyme

##### MBIC and MBEC were determined by using spectrophotometric microtiter plate assay

At this experiment, biofilm-forming *P. aeruginosa* (ATCC 35659) isolate was taken from the Microbiology lab in Tanta hospital. Purified α-amylase from isolated *B. cereus* as well as commercial α-amylase from *B. amyloliquefaciens* (Sigma Aldrich, Germany) were used to evaluate the antibiofilm activity via determining the MBIC and MBEC values. Overnight culture of *P. aeruginosa* bacterial cells was inoculated in TSB media at 0.5McFarland, then incubated for 5 days in 96 well- polystyrene microtiter plate to allow mature biofilm to grow for 5 days, culture media was changed every 48 h to maintain cell viability, then wells OD was analyzed by ELISA Microplate Reader (PowerWaveX, Bio-Tek, USA) at wavelength 610 nm to determine absorbance that reflects biofilm formation capability. Plates with bacterial biofilm served as positive control and plates with culture media only served as negative control. An aliquot (50 μl) of twofold serially diluted purified and commercial α-amylase enzymes with starting concentration of 500 and 400 µg/ml, respectively were introduced to microtiter plate wells and incubated for 48 h at 37 °C. After incubation supernatant was discarded and each well washed 3 times with 200 μl of sterile distilled water and plates allowed to dry for 20 min and stained with crystal violet at room temp for 10 min, then washed 3 times with sterile distilled water to get rid of excess stain and then absorbance was measured at 610 nm using a spectrophotometer (Biotech instruments, USA). MBIC considered as minimum concentration of enzyme which inhibit biofilm formation with no longer ability of microorganism to form more biofilm in wells and no more increase in OD values of test wells, and MBEC is considered as the minimum concentration which remove 98% of formed biofilm from the bottom of the treated wells. Experiments were performed in triplicates and means of values were calculated [27].

#### Biofilm formation and inhibition assay

The biofilm formation capability of the *P. aeruginosa* was evaluated by using microscopic and spectrophotometric analysis techniques utilizing 96-well microtiter culture plates. Briefly, overnight culture of *P. aeruginosa* (ATCC 35659) strain adjusted at 0.5 McFarland (1.5 × 10^8^ CFU ml^−1^) was used to inoculate LB broth (pH 7) which is poured in microtiter plate wells and then subjected to incubation at 37 °C for 5 days to allow biofilm to grow. After incubation of plates, the wells were washed three times with sterile PBS and air dried at room temperature for 15 min. After that, the wells had been stained with sterile crystal violet dye (1% w/v) and incubated for 10 min to allow good staining of the biofilm in wells. After that, wells were washed again with sterile distilled water to remove excess stain and kept at room temp for 20 min to dry. Finally, wells were destained with absolute ethanol 99% and the optical activity was assessed at 610 nm by a spectrophotometer (SHIMADZU, UV-2450, Japan) [27].

The effect of α-amylase as antibiofilm agent was evaluated by spectrophotometric assays and laser scanning microscopy observations. The volume of commercial and extracted α-amylase (30 μl) from *B. cereus* and *B. amyloliquefaciens* at their MBEC levels was added to 170 μl of Luria Bertani broth media at pH 7 containing bacterial suspensions incubated previously at 37 °C for 48 h at concentration of 0.5 McFarland (1.5*10^8^ cfu/ml). Wells with bacterial biofilms without enzyme served as positive controls and wells with culture media served as negative control. The plates were been incubated at 37 °C for 24 h and crystal violet staining was done as mentioned in the previous steps [27, 28].

$$ {\text{percentage of biofilm inhibition }} = {\text{Optical density of control sample {-} optical density of test sample/optical density of control sample}} \times {100} $$

#### CLSM for analysis of biofilm thickness and viability percentage

*P. aeruginosa* biofilm was allowed to grow by incubation at 37 °C of bacterial culture (0.5McFarland) for 5 days and samples taken were served as positive control. Tested biofilm samples were allowed to grow for 48 h after treatment with extracted and commercial α-amylase. Eight-well chamber slides were utilized for growth of bacterial biofilm. After formation of biofilm, positive control sample was stained by live florescent dye acridine orange AO (0.01 mg/ml, sigma Aldrich, Germany; excitation wavelength 488 nm/emission wavelength 515 nm) to stain live cells and tested samples were stained by mixture of freshly prepared acridine orange (vol. 5 µl) and propidium iodide PI (vol. 5 µl) of 0.01 m g/ml, (Sigma Aldrich, Germany; excitation wavelength 488 nm/emission wavelength 630 nm) dyes for visualization of live/dead cells following the manufacturer instructions using (dual fluorescent staining method). After staining, slides remained in darkness for 15 min at room temperature and then visualized using confocal laser scanning microscope (Leica DMi8, Germany). Experiment was done in triplicates and mean of values was selected.

AO is a cell-permeating fluorescent dye that beams green fluorescence when bound to dsDNA. PI is a fluorescent dye that is cell membrane impermeable and mostly prohibited from live cells, so, live cells will give out green colour, while dead cells with damaged membranes will give out red colour [29].

$$ {\text{Percent of thickness inhibition = }}{{{\text{thickness of control sample (}}\mu {\text{m) - thickness of test sample (}}\mu {\text{m)}}} \mathord{\left/ {\vphantom {{{\text{thickness of control sample (}}\mu {\text{m) - thickness of test sample (}}\mu {\text{m)}}} {{\text{thickness}}}}} \right. \kern-0pt} {{\text{thickness}}}}{\text{of}}\,{\text{controlsample (}}\mu {\text{m)}} \times {100} $$

Images obtained from scanning microscope were analyzed by Fiji (image) biofilm analyzer software version 1.53t 2022 [30] to determine live/dead cells percentage [31, 32].

#### Statistical analysis of results

The variability degree of the results obtained was calculated as mean and based on 3 independent values (*n* = 3). Statistical analysis of the data had been performed by one-way ANOVA test. Expert design software (version. 7) was used to analyze the results [31, 32].

## Results and discussion

### Isolation and identification of amylase-producing bacteria

**Fig. 1:**
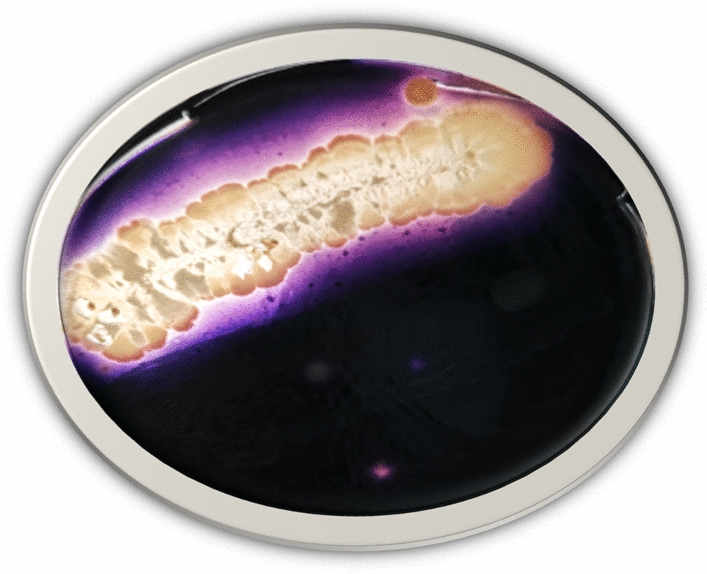
Starch hydrolysis test of isolated *Bacillus* spp. showing halo zone around colonies

In the present study, a total of 12 distinct isolates of genus *Bacillus* were recovered from the collected soil samples obtained from Sharqia governorate in Egypt. Preliminary identification as *Bacillus* was done based on the colonial morphology, as well as the Gram-staining technique. On soluble starch agar media, the amylase-producing capacity of 7 out of 12 (58.33%) *Bacillus* isolates was confirmed showing different measurements of halo zones ranging between 10 and 17 mm. Among these positive amylase-producing isolates, the one (isolate no. 7) with the highest zone of hydrolysis was selected for further research as shown in Fig. 1. These findings were in agreement with the study of Hallol et al. [33] where the recorded inhibition zones were 15 mm or more for the test bacterial isolates. Additionally, they reported that the best isolate; *Bacillus paramycoides* produced 177.12 U/mg of α-amylase enzyme according to the quantitative DNS assay. Basically, the later method depends on the breakdown of starch into glucose and maltose by α-amylase enzyme, and so the amount of α-amylase is related to the amount of sugar produced from maltose standard curve. In the current work, it was found that isolate no. 7 produced 135 U/ml of α-amylase enzyme.

**Fig. 2:**
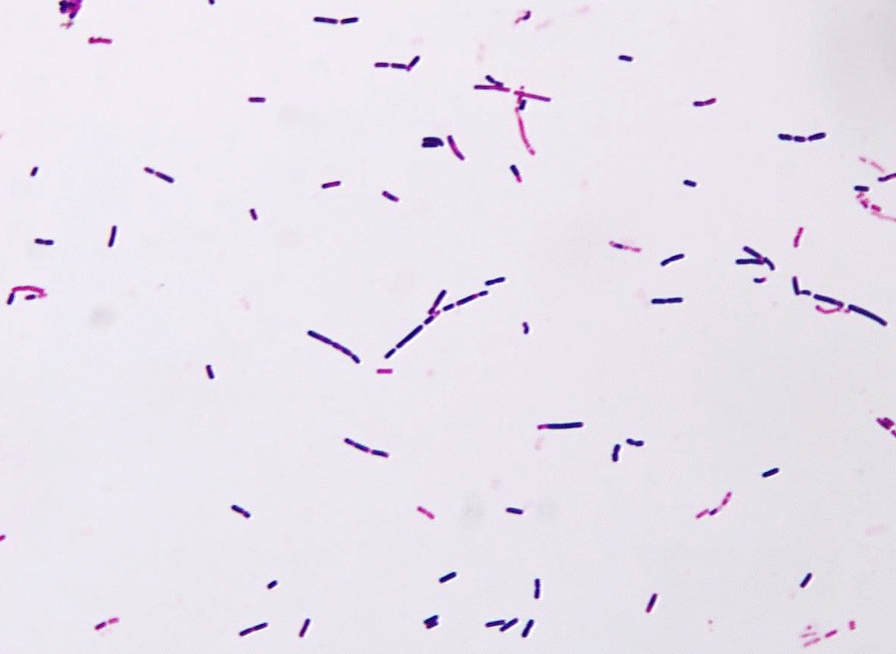
Microscopic visualization of *Bacillus cereus* examined under light microscope

**Fig. 3:**
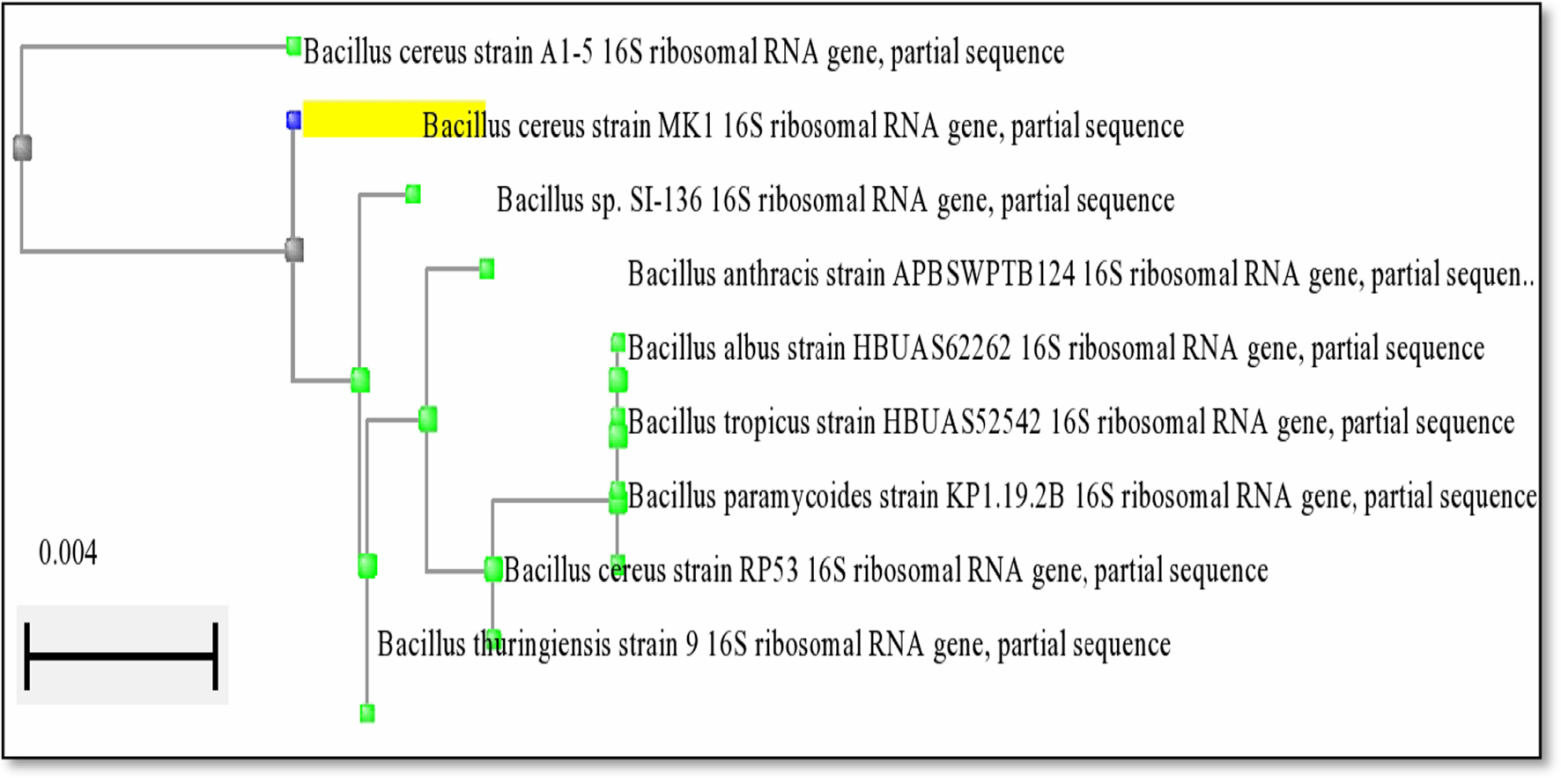
Phylogenetic analysis of *B. cereus* isolate depending on 16 s rRNA sequence homology using BLAST. It was noticed that *B. cereus* A1-5 was the closest strain to the test isolated species

Therefore, the selected *Bacillus* isolate was further subjected to identification to the species level using standard biochemical tests as described in Bergey’s Manual of Bacteriology as well as the 16S rRNA. Based on the results, it was identified as *Bacillus cereus* depending on the biochemical characters presented in Table 1 and Fig. 2. Furthermore, the comparison between the 16S ribosomal RNA gene nucleotide sequence (395 base pairs) of the isolated bacteria with other 16S ribosomal RNA gene nucleotide sequences of closely associated isolates at NCBI database discovered that this isolate shared 99% sequence closeness with *B. cereus* strain A1-5. Figure 3 displayed the neighbor-joining phylogeny showing that the test bacterium belongs to *Bacillus genus family* and strongly linked to *B. cereus* strain MK 1 and *B. Cereus* strain A1-5. The obtained *B. cereus* nucleotide sequence had been deposited in the Gene Bank database and recorded under the accession number (OP811897) (http://ncbi.nlm.nih.gov/OP811897) [34, 35].

**Table 1:** Morphological and biochemical characters of *Bacillus* isolate

Feature	Results
Characters on nutrient agar	Creamy white colonies
Gram-staining/morphology under microscope	Single rod-shaped or short-chained bacilli with square ends
Catalase	Positive
Citrate	Positive
Gelatin hydrolysis	Positive
Hemolysis	Positive
Methyl red	Positive
Indole	Positive
VP	Positive
Oxidase	Negative
Carbohydrate fermentation	Positive
Nitrate reduction	Variable
Motility	Motile
Casein hydrolysis	Positive
Esculin hydrolysis	Positive

### Determination of optimal basal conditions for α-amylase production (un-optimized conditions) by OFAT protocol

#### Effect of various fermentation media on α-amylase yield

**Fig. 4:**
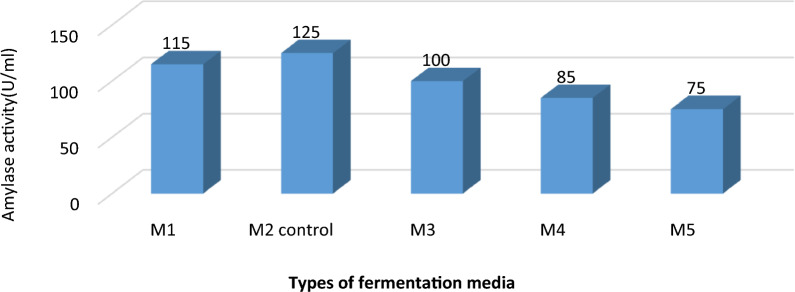
Effect of different fermentation media on α-amylase activity

Results revealed that the optimum production of α-amylase enzyme was obtained when the selected organism was grown in M2 medium which was used as a control basal medium for our OFAT protocol as presented in Fig. 4. Using the later medium resulted in an enzymatic production which was 1.6-fold higher than using M5 and 1.4-fold higher than using M4 medium. Hence, the optimum production was related to the existence of additive nutrients and metal ions ex: (NH4)_2_SO4 and MgSO4 which are essential for growth of the test bacteria. On the contrary, some metal ions like BaCl2 and MnCl2 decreased the microbial amylase production as shown in metal ions experiments and Media; M4 and M5. Our results were close to other findings [36].

#### Role of various fermentation time on α-amylase production

**Fig. 5:**
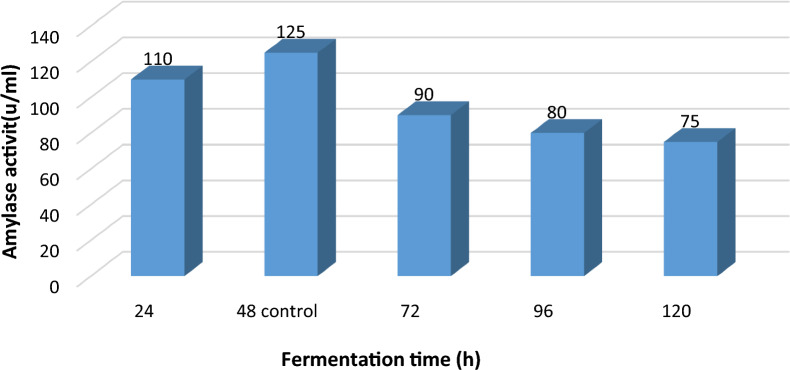
Effect of different fermentation time on α- amylase activity

According to the findings in Fig. 5, the produced enzyme had the greatest activity at 48 h (125U/ml) and the least activity at 120 h (75 U/ml). Although increasing the incubation period could result in a decrease in activity, it did not result in an increase in enzyme production. This is because the cells have started to degrade and their ability to assimilate enzymes has decreased. Depletion of nutrients and the onset of the organism's death phase could possibly be to blame. The findings showed that denaturation of the enzyme brought on by interactions with numerous other elements in the medium may be the reason of a drop in enzyme production after 48 h of incubation [1, 16]. Additionally, Rakaz et al. [37] mentioned that the activity of α- amylase from *B. cereus* was 0.122 U/ml/min at 24 h. Moreover, they reported that increasing the incubation time to more than 24 h resulted in a rapid decrease in the enzymatic activity.

#### Impact of different pH on α-amylase yield:

**Fig. 6:**
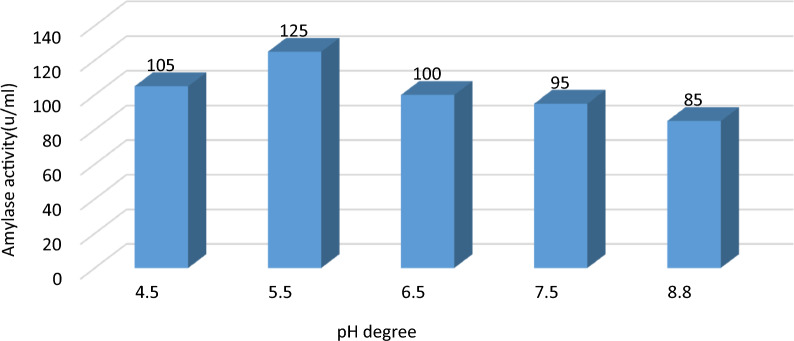
Effect of different pH on α-amylase activity

The pH of the production medium impacts enzyme synthesis since it is crucial for microbial development. The enzyme activity was measured at pH levels ranging from 4.5 to 8.5, with the optimal incubation time set at 48 h and adjusted with HCL (1 M) and NaOH (1 M). As indicated in Fig. 6, the highest α-amylase production was discovered at pH 5.5 (125 U/ml), whereas the lowest production was found at pH 8.8 (85 U/ml). Further changes in pH caused a progressive decrease in the activity of α-amylase. Additionally, the activity is diminished when the pH was altered below or above the optimal threshold [1, 16]. Previous research on α-amylase production discovered that pH had a higher impact on α-amylase production. Rakaz et. al. [37] reported that microbial α-amylase production from *B. cereus* MCM B-326 and AS2 decreased by 40% when pH was adjusted to more than 8.

#### Impact of different temperature on α-amylase production:

**Fig. 7:**
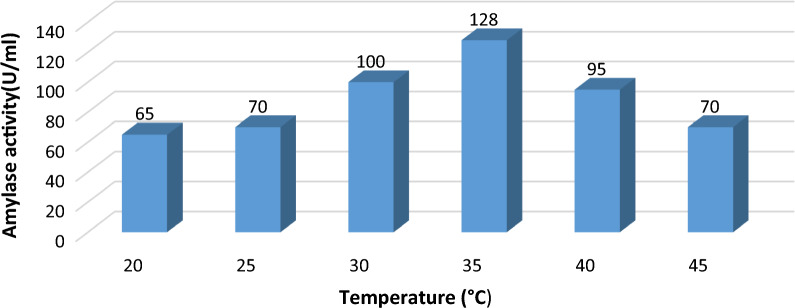
Impact of various temperature degrees on amylase activity

Alpha-amylase yield was measured at temperatures varied from 20 °C to 45 °C, with a pH of 5.5 and a time span of 48 h. The enzyme production was found to be maximal at 35 °C (128 U/ml) and the lowest activity was at 45 °C (70U/ml) followed by 40 °C (95 U/ml) as shown in Fig. 7**.**

Vijayaraghavan et. al [30] had shown that enzyme activity produced by *Bacillus subtilis* and *B. cereus* recorded the maximum activity at 45 °C. Similarly, Rakaz et. al. [37] reported that incubating *B. cereus* at 45 °C was optimal for α-amylase production. Additionally, optimum temperature of 65 °C was mentioned for α-amylase enzyme produced from other *Bacillus* species like *B. licheniformis* Rakaz et. al. [37]. Interestingly, *B. marini's* previous report on optimum α-amylase production at 40 °C revealed that as the temperature increased, the enzyme activity was gradually decreased. This could be because high temperatures inhibit bacterial growth, because temperature has an effect on bacterial metabolism and cell viability. The enzyme's production decreased as the temperature rises. It was discovered to be maximal at 35 °C, indicating a one-to-one relation between enzyme production and biomass, this is giving the clue that enzyme production is growth-dependent. Higher (45 °C) or lower (20 °C) temperature degrees reduced the enzyme production by ~ 1.8 fold [1, 16]. Saha and Mazumdar et. al [13] reported that OFAT protocol showed activity approximately 3.9-fold higher as compared to unoptimized conditions.

#### Impact of different carbon sources on α-amylase yield:

**Fig. 8:**
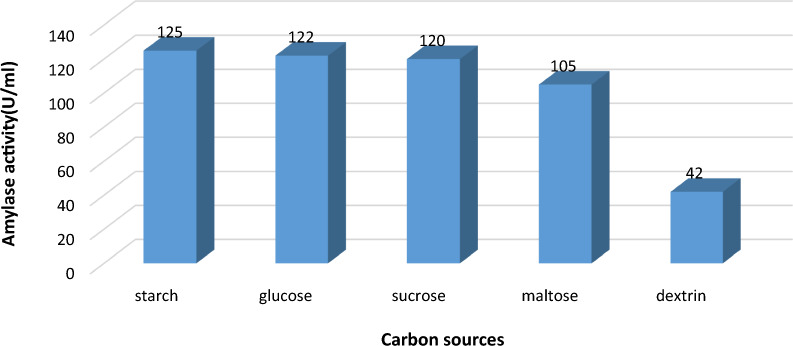
Effect of different carbon sources on α-amylase activity

It was discovered how carbon sources affected the synthesis of α-amylase. The best carbon source among the several ones examined, as shown in Fig. 8, was starch (125 U/ml), followed by glucose (122 U/ml) and sucrose (120 U/ml). Dextrin, on the other hand, was discovered to be an inhibitor of α-amylase synthesis since it had lowered α-amylase activity (42 U/ml) by threefold when used. Another set of research results revealed that different types of organisms have different capacities for using carbon sources to manufacture the bacterial enzymes [3, 15, 17]. In contrast to our finding, Abdel-Nabey and Farag [38] obtained maximum α-amylase activity from *B. lichineformis* AH214 in presence of maltose followed by glucose while lactose gave the lowest activity. Sreekanth et. al [15] suggested that the ability to use a carbon source to produce enzymes varied according to the type of the organism.

#### Effect of different nitrogen source on α-amylase production:

**Fig. 9:**
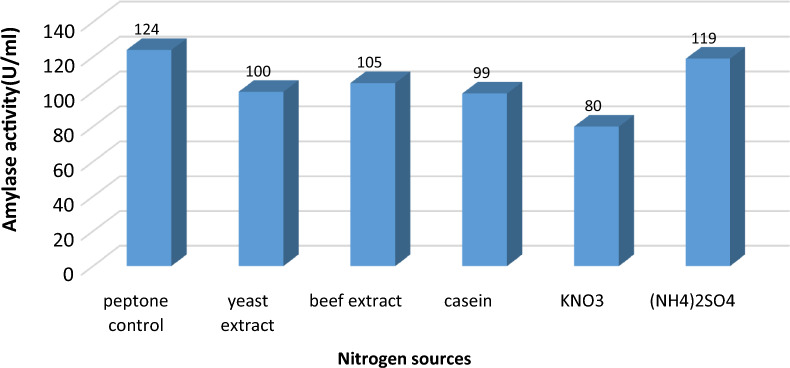
Effect of different nitrogen sources on amylase activity

Effect of different nitrogen source on α-amylase production was also evaluated because organic and inorganic nitrogen sources play a vital role in α-amylase production. Amylase showed the best activity upon utilization of peptone as a nitrogen source (124U/ml) followed by (NH4)_2_SO4 (119 U/ml). Casein and KNO3 reduced α-amylase activity by 1.2- and 1.5-fold (99 U/ml, 80 U/ml), respectively when tested as nitrogen sources. The capability of utilization of fermentation media with different nitrogen sources affects the enzyme production which differs from organism to another as presented in Fig. 9. Hallol et al. [33] reported that peptone had a significant impact of the production of α-amylase. Additionally, Simair et. al [8] suggested that the beef extract was the best N-source for α-amylase production by *Bacillus* sp. BCC 01–50. Also, Kumar et. al [19] reported that each organism or strain has its own special condition for maximum enzyme production. Other researcher findings noticed that each microorganism has its own specific culture conditions for maximum α-amylase production [3, 17, 19].

#### Effect of different metal ions on α-amylase production

**Fig. 10:**
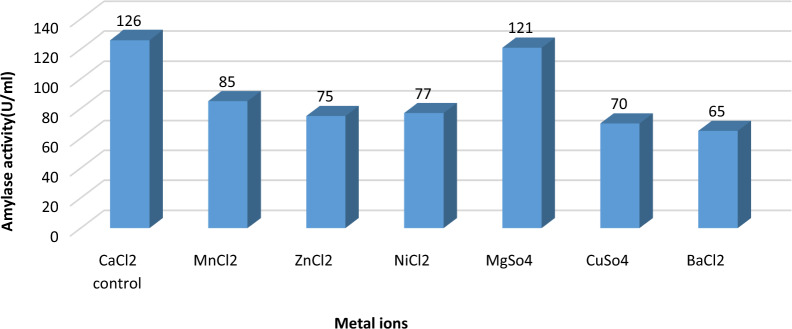
Effect of various metal ions on α-amylase production

In this experiment, the effect of various types of metal ions on enzyme production was assessed at final concentrations of 10 mM. The maximum yield of α- amylase enzyme was obtained by utilization of CaCl2 (126 U/ml) followed by MgSO4 (121 U/ml), however the lowest production obtained by utilization of BaCl2 (65U/ml) followed by CuSO_4_ (70 U/ml). Enzyme activity was inhibited by 55% and 51% after using CuSO4 and BaCl2, respectively as metal ion source as shown in Fig. 10. Results were confirmed by statistical optimization of α-amylase enzyme [9, 33, 37]. Lin et. al [39] suggested that enzyme was activated with Ca^2+^, while it was inhibited in the presence of Hg^2+^. The effect of various metal ions on purified α-amylase activity was reported by Mamo and Gessesse et. al [40], and Fincan et. al [41].

#### Optimization of α-amylase production by statistical design Plackett–Burman

Plackett–Burman design (PBD), followed by central composite design (CCD), was used in order to optimize the critical parameters needed for maximum production of α-amylase by *B. cereus* isolate no. 7. Hallol et al. [33] previously used PBD to determine the factors that could affect α-amylase production by *B. paramycoides* MS009. In the present work, PBD was used to screen 11 independent variables as shown in Table 2: A, starch; B, glucose; C, lactose; D, yeast extract; E, peptone; F, (NH4)_2_SO4; G, CaCl2; H, KCL; J,MgSO4; K, CuSO4; L, tween 80 had been examined at 2 levels: low level (− 1) and high level (+ 1) and studied for their qualitative effect on α-amylase production giving 12 run trials and the success of the design is analyzed by ANOVA as shown in Table 3. Amylase activity (U/ml) was calculated from sugar standard curve and the PBD showed the predicted value by establishing first order polynomial equation to evaluate the production of α-amylase as following:

$$ \begin{aligned} \alpha {\text{ - amylase activity }}\left( {\text{U/ml}} \right) & = {128}{\text{.25 + 13}}{\text{.08*}{\text{glucose}}} + {12}{\text{.25*}}{\text{peptone + 14}}{\text{.48*}}\left( {{\text{NH4}}} \right)_{{2}} {\text{SO}}_{{4}} \\ & - {16}{\text{.75*}{\text{KCL + 12}}}{\text{.42*}{\text{MgSO}}}_{{4}} {-} {{4}}{\text{.25*}{\text{CuSO}}}_{{4}} { + 7}{\text{.25*}{\text{tween 80}}} \\ \end{aligned} $$

**Table 2:** Test factors and levels used in optimization of α-amylase production by PBD model

Plackett–Burman design matrix																								
Run	Factor 1		Factor 2		Factor 3		Factor 4		Factor 5		Factor 6		Factor 7		Factor 8		Factor 9		Factor 10		Factor 11		Response 1	Response 2
	A:Starch g/l		B:Glucose g/l		C:Lactose g/l		D:yeast extract g/l		E:peptone g/l		F:(NH_4_)_2_ SO_4_ g/l		G:CaCl_2_ g/l		H:KCl g/l		J:MgSO_4_ g/l		K:CuSO_4_ g/l		L:Tween 80 g/l		predicted α-amylase activity	calculated α-amylase activity
																							U/ml	U/ml
**1**	− **1**	**5**	**1**	**5**	**1**	**5**	− **1**	**5**	**1**	**10**	**1**	**10**	**1**	**0.05**	− **1**	**0**	− **1**	**0**	− **1**	**0**	**1**	**1**	**195**	**195**
2	1	15	1	5	1	5	− 1	5	− 1	5	− 1	5	1	0.05	− 1	0	1	0.25	1	0.05	− 1	0	137	137
3	− 1	5	− 1	0	− 1	0	1	10	− 1	5	1	10	1	0.05	− 1	0	1	0.25	1	0.05	1	1	152	152
4	1	15	1	5	− 1	0	1	10	1	10	1	10	− 1	0	− 1	0	− 1	0	1	0.05	− 1	0	150	142
5	1	15	− 1	0	1	5	1	10	− 1	5	1	10	1	0.05	1	0.05	− 1	0	− 1	0	− 1	0	88	88
6	− 1	5	1	5	− 1	0	1	10	1	10	− 1	5	1	0.05	1	0.05	1	0.25	− 1	0	− 1	0	134	134
7	− 1	5	1	5	1	5	1	10	− 1	5	− 1	5	− 1	0	1	0.05	− 1	0	1	0.05	1	1	90	90
8	1	15	1	5	− 1	0	− 1	5	− 1	5	1	10	− 1	0	1	0.05	1	0.25	− 1	0	1	1	142	137
9	− 1	5	− 1	0	1	5	− 1	5	1	10	1	10	− 1	0	1	0.05	1	0.25	1	0.05	− 1	0	130	130
10	1	15	− 1	0	− 1	0	− 1	5	1	10	− 1	5	1	0.05	1	0.05	− 1	0	1	0.05	1	1	85	76
11	− 1	5	− 1	0	− 1	0	− 1	5	− 1	5	− 1	5	− 1	0	− 1	0	− 1	0	− 1	0	− 1	0	87	87
12	1	15	− 1	0	1	5	1	10	1	10	− 1	5	− 1	0	− 1	0	1	0.25	− 1	0	1	1	149	149

For each factor, the first column presented the testing levels and the second column provided the actual concentration of the compound

**Table 3:** Analysis of variance (ANOVA) of PBD model

Source	Sum of square	df	Mean square	F value	p-value	
					Prob > F	
Model	12471.25	7	1781.607143	17.42403074	0.0075	Significant
B-glucose	2054.083333	1	2054.083333	20.08883456	0.0110	
E-peptone	1800.75	1	1800.75	17.61124694	0.0137	
F-(NH4)_2_SO_4_	2552.083333	1	2552.083333	24.9592502	0.0075	
H-KCl2	3366.75	1	3366.75	32.92665037	0.0046	
J-MgSO4	1850.083333	1	1850.083333	18.09372453	0.0131	
K-CuSO4	216.75	1	216.75	2.119804401	0.2191	
L-Tween 80	630.75	1	630.75	6.168704156	0.0679	
Residual	409	4	102.25			
Cor Total	12880.25	11				
			R-Squared	0.968245958		
			Adj R-Squared	0.912676384		
			Pred R-Squared	0.714213622		

**Fig. 11:**
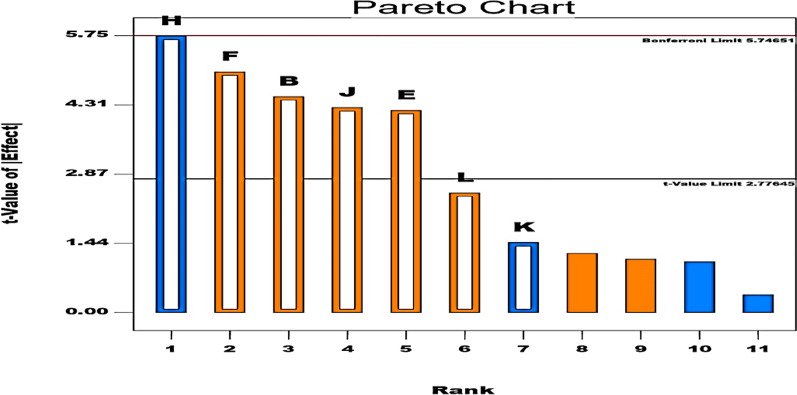
Pareto chart showing positive significant and negative significant factors affecting α-amylase production by *Bacillus cereus*. B = glucose *, E = peptone*, F = (NH4)2 SO4*, H = KCl*, J = MgSO4*, K = CuSO4, and L = tween 80. * Refers to significant parameters

Data in Table 2 revealed that interaction between 11 factors gave wide variation in α-amylase production from 85 to 195 U/ml with the highest activity in the run (number. 1) recording an activity of 195 U/ml resulting in 1.5-fold increase in the activity in comparison with un-optimized media composition (M2:125 U/ml). Pareto chart (Fig. 11**)** provides that of the 11 tested factor, there are positive significant factors affecting α-amylase production (MgSO4, (NH4)_2_SO4, glucose and peptone presented as orange-colored bars and negative significant factors affecting α-amylase production appeared as blue-colored bars (KCL and CuSO4). According to Sundarram and Murthy [3] glucose showed the most promotive effect on *B. cereus* α-amylase production. Saha et al. [13], Wang et al. [42] and Naranchimeg et al [43] reported the enhancement effect of Ca ^2+^ and peptone on the production of *Bacillus* α-amylase. Maltose was reported by Elmansy et al. [44] as a carbon source for *Bacillus sp.* amylase production. Glucose and sucrose were found to come after starch as a carbon source for the production of *Enterobacter hormaechei* and *Bacillus cereus* amylases [45].

Glucose and sucrose were found to come after starch as a carbon source for *cereus* amylases. The success of the design was statistically assessed by ANOVA test as shown in Table 3. The model F-value of 17.42 revealed significance of the model. Squared regression coefficient R^2^ is 0.9682 meaning that 96.8% of the results could be explained by the design. The closeness between the predicted R^2^ (0.7142) and adjective R^2^ (0.9127) emphasizes the success of the design [13, 18].

#### Optimization of α-amylase production by response surface methodology central composite design (CCD)

Four factors A, (NH4)_2_SO4; B, glucose; C, MgSO4; D, peptone revealed highest α-amylase activity from PBD were screened with 5 levels: (0), (+ 1), (− 1), (+ 2), (− 2) and were optimized by CCD for their quantitative effect on α-amylase production giving 30 trial runs as shown in Table 4. Media composition in the run no.1 which revealed the highest α-amylase activity in PBD was selected as control media for CCD experiments. The success of the design was analyzed by ANOVA as shown in Table 5. From the PBD results, it was concluded that (NH4)_2_SO4, glucose, peptone and MgSO_4_ have the highest positive effect on enzyme yield. The optimum enzyme production was shown in the run (number. 13) with activity (250U/ml) which is twofold more than un-optimized media (M2:125 U/ml). The highest α-amylase activity was obtained with media composed of g/l: starch, 5; glucose, 7.5; lactose, 5; yeast extract, 5; peptone, 12.5; (NH4)_2_SO4, 12.5; CaCl2, 0.05; MgSO4, 0.75; tween 80, 1.

**Table 4:** Test factors and levels used for optimization α-amylase by CCD model showing the most significant factors that affected the production

Central composite design matrix										
Run	Factor 1	Factor 2			Factor 3		Factor 4		Response 1	Response 2
	A:(NH_4_)_2_SO4 g/l	B:glucose g/l			C:MgSO_4_ g/l		D:Peptone g/l		Predicted α-mylase activity	Calculated α-amylase activity
									U/ml	U/ml
1	− 2	7.5	0	7.5	0	0.75	0	12.5	160	155
2	− 1	10	− 1	5	− 1	0.5	1	15	158	158
3	1	15	− 1	5	− 1	0.5	1	15	195	190
4	1	15	1	10	− 1	0.5	1	15	187.08	179
5	− 1	10	− 1	5	− 1	0.5	− 1	10	159.83	162
6	1	15	− 1	5	1	1	− 1	10	181.5	190
7	1	15	1	10	1	1	− 1	10	164.42	160
8	0	12.5	0	7.5	0	0.75	0	12.5	249	240
9	0	12.5	0	7.5	0	0.75	0	12.5	249	240
10	− 1	10	− 1	5	1	1	1	15	161	155
11	0	12.5	0	7.5	− 2	0.25	0	12.5	161	157
12	− 1	10	1	10	− 1	0.5	1	15	159.83	163
**13**	**0**	**12.5**	**0**	**7.5**	**0**	**0.75**	**0**	**12.5**	**249**	**250**
14	0	12.5	0	7.5	0	0.75	0	12.5	249	250
15	1	15	1	10	1	1	1	15	181.5	180
16	1	15	− 1	5	1	1	1	15	196	196
17	− 1	10	1	10	− 1	0.5	− 1	10	166.25	165
18	1	15	1	10	− 1	0.5	− 1	10	135	135
19	0	12.5	0	7.5	0	0.75	0	12.5	249	238
20	− 1	10	1	10	1	1	− 1	10	195	195
21	− 1	10	− 1	5	1	1	− 1	10	197.75	202
22	− 1	10	1	10	1	1	1	15	135	140
23	0	12.5	0	7.5	0	0.75	2	17.5	146	146
24	0	12.5	2	12.5	0	0.75	0	12.5	184	188
25	0	12.5	− 2	2.5	0	0.75	0	12.5	197	197
26	0	12.5	0	7.5	0	0.75	− 2	7.5	156.08	162
27	0	12.5	0	7.5	0	0.75	0	12.5	249	235
28	1	15	− 1	5	− 1	0.5	− 1	10	146	146
29	0	12.5	0	7.5	2	1.25	0	12.5	189.08	200
30	2	17.5	0	7.5	0	0.75	0	12.5	199	199

For each factor, the first column presented the testing levels and the second column provided the actual concentration of the compound

**Table 5:** Analysis of variance (ANOVA) of CDD for α-amylase optimization

Source	Sum of squares	df	Mean square	F value	P-value	
					Prob > F	
Model	38186.51745	14	2727.60839	70.054732	< 0.0001	Significant
A-(NH4)_2_SO4	724.2410667	1	724.2410667	18.60109905	0.0006	
B-glucose	392.0416667	1	392.0416667	10.06903117	0.0063	
C-MgSO4	1084.60815	1	1084.60815	27.85661371	< 0.0001	
D-Peptone	2.34375	1	2.34375	0.060195877	0.8095	
AB	56.25	1	56.25	1.444701039	0.2480	
AC	15.015625	1	15.015625	0.385654916	0.5439	
AD	3530.142225	1	3530.142225	90.66666917	< 0.0001	
BC	154.132225	1	154.132225	3.958666411	0.0652	
BD	30.747025	1	30.747025	0.789693493	0.3882	
CD	1560.25	1	1560.25	40.07279638	< 0.0001	
A^2	8940.010671	1	8940.010671	229.6114259	< 0.0001	
B^2	6423.9021	1	6423.9021	164.9887651	< 0.0001	
C^2	10078.38107	1	10078.38107	258.8488464	< 0.0001	
D^2	17375.06679	1	17375.06679	446.2538141	< 0.0001	
Residual	584.0308667	15	38.93539111			
Lack of fit	584.0308667	10	58.40308667			
Pure error	0	5	0			
Cor total	38770.54832	29				

The enzyme yield was calculated from the following equation:

$$ \begin{aligned}  {\text{Amylase activity (}}{{\text{U}} \mathord{\left/ {\vphantom {{\text{U}} {\text{ml)}}}} \right. \kern-0pt} {\text{ml)}}} & = 249 + {\text{5.49*}}({\text{NH}}_{{4}} )_{2} {\text{SO}}_{4}-{\text{4.04*}}{\text{glucose}} \\ & + {\text{6.72*}}{\text{MgSO}}_{{4}} + {\text{0.31*}}{\text{peptone}}-{\text{1087*}}{{(\text{NH}}}_{4} )_{2} {\text{SO}}_{4}{\text{*}}{\text{glucose}} \\ & + {0}{\text{.97*}}{{(\text{NH}}}_{{4}} {)}_{{2}} {\text{SO}}_{{4}}{\text{*}} {\text{MgSO}}_{{4}} { + 14}{\text{.84*}}{(}{\text{NH}}_{{4}} {)}_{{2}} {\text{SO}}_{{4}} {\text{*}}{\text{peptone}} \\ & - {3}{\text{.10*}}{\text{MgSO}}_{4} - {\text{1.39*}}{\text{glucose*}}{\text{peptone}}-{9}{\text{.87*}}{{\text{MgSO}}}_{4} {\text{*}}{\text{peptone}} \\ & - {10}{\text{.05*}}{{(\text{NH}}}_{4} )_{2} {\text{SO}}_{4}^{2}-{\text{15.30*}}{\text{glucose}}^{2} - {\text{19.17*}}{\text{MgSO}}_{{4}}^{2} - {\text{25.17*}}{\text{peptone}}^{2} \\ \end{aligned} $$

The success of the design is statistically analyzed as shown in Table. 5 by (ANOVA). The model F- value of 70.05 reveals that the model is significant, R2 is 0.9849 meaning that 98.49 of the results can be explained by the design. The closeness between the values of predicted R^2^ (0.9132) and Adjective R^2^ (0.9709) emphasizes the success of the design [18].

**Fig. 12:**
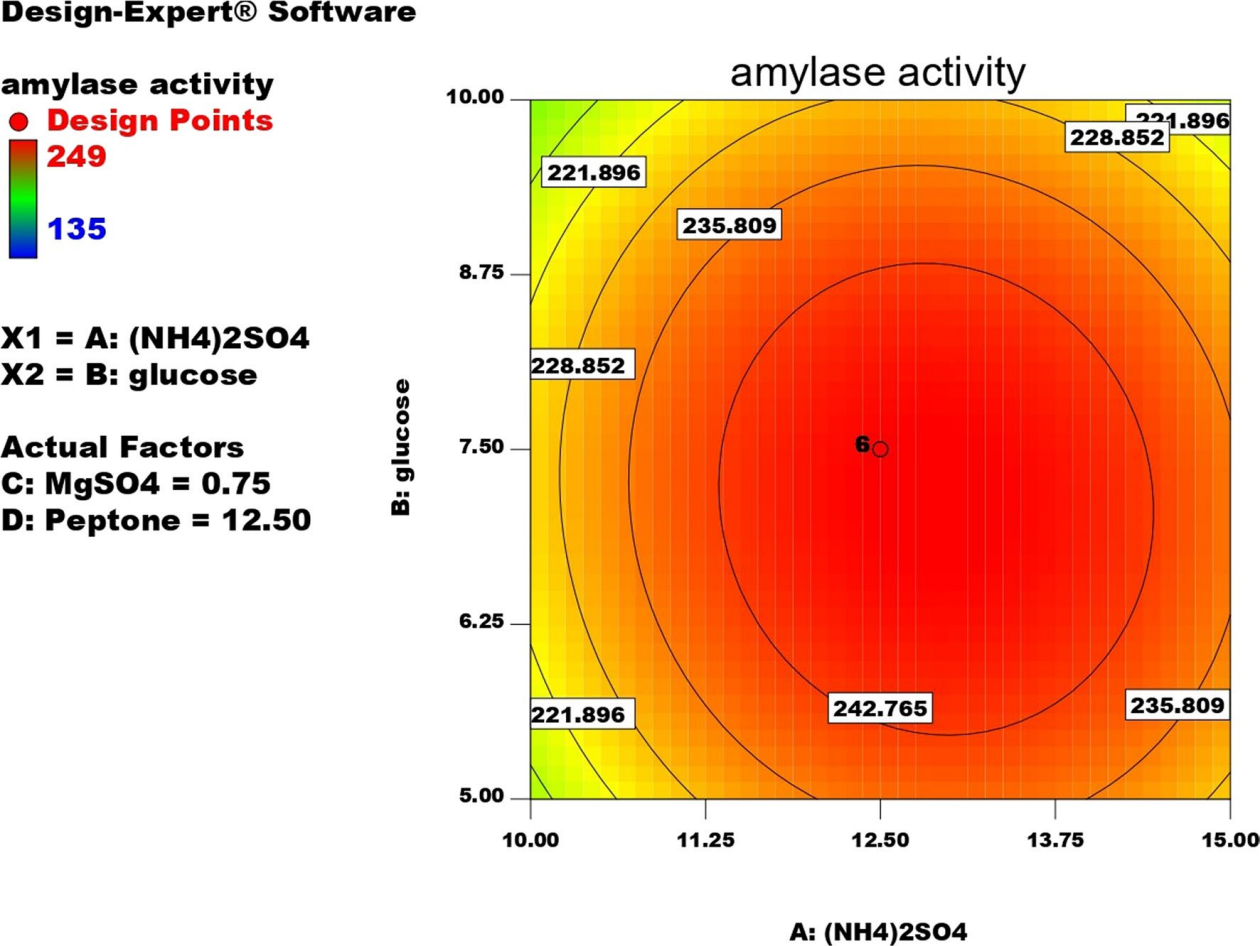
Contour plot shows interaction between glucose and (NH4)_2_SO4

**Fig. 13:**
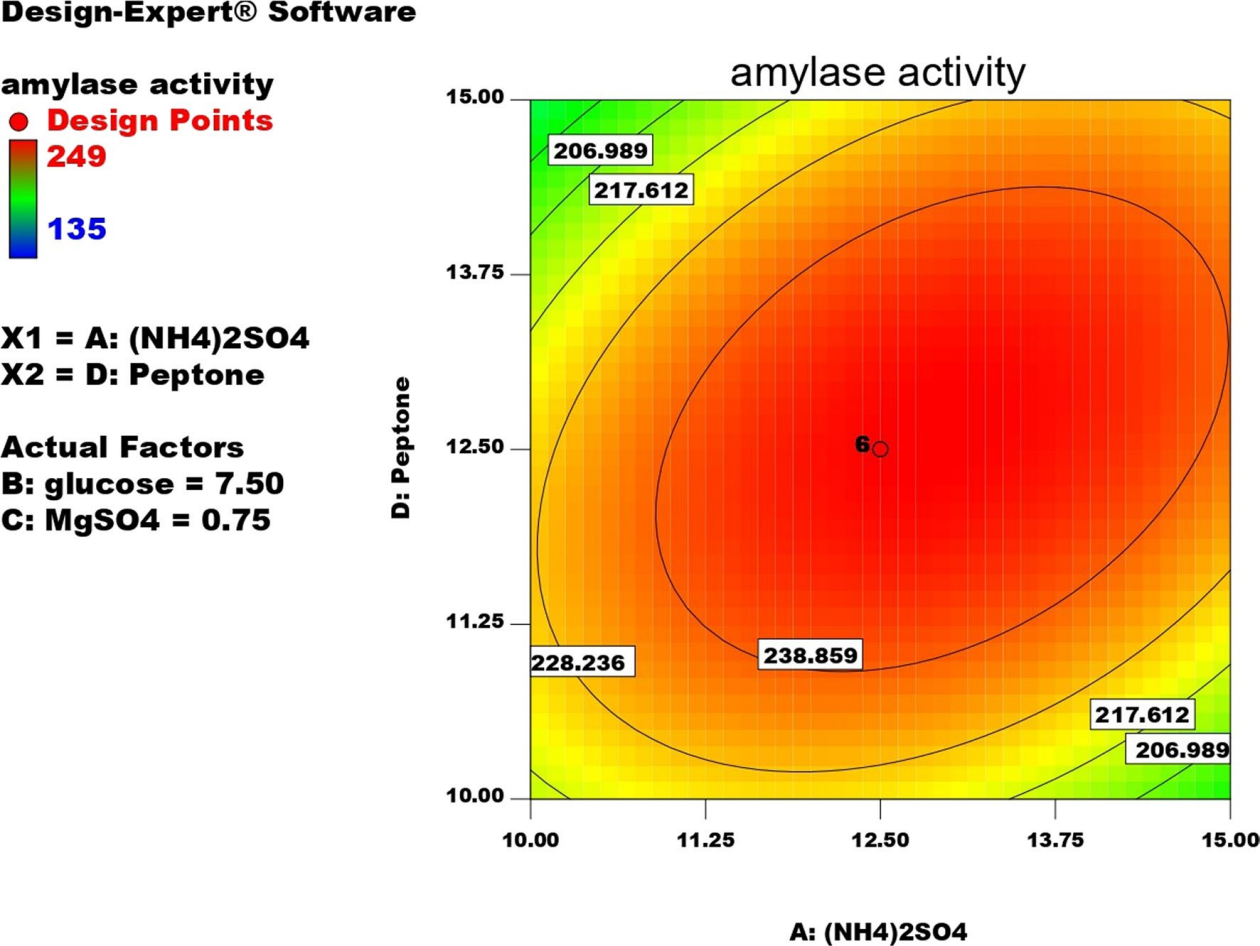
Contour plot shows interaction between peptone VS (NH4)_2_SO4

**Fig. 14:**
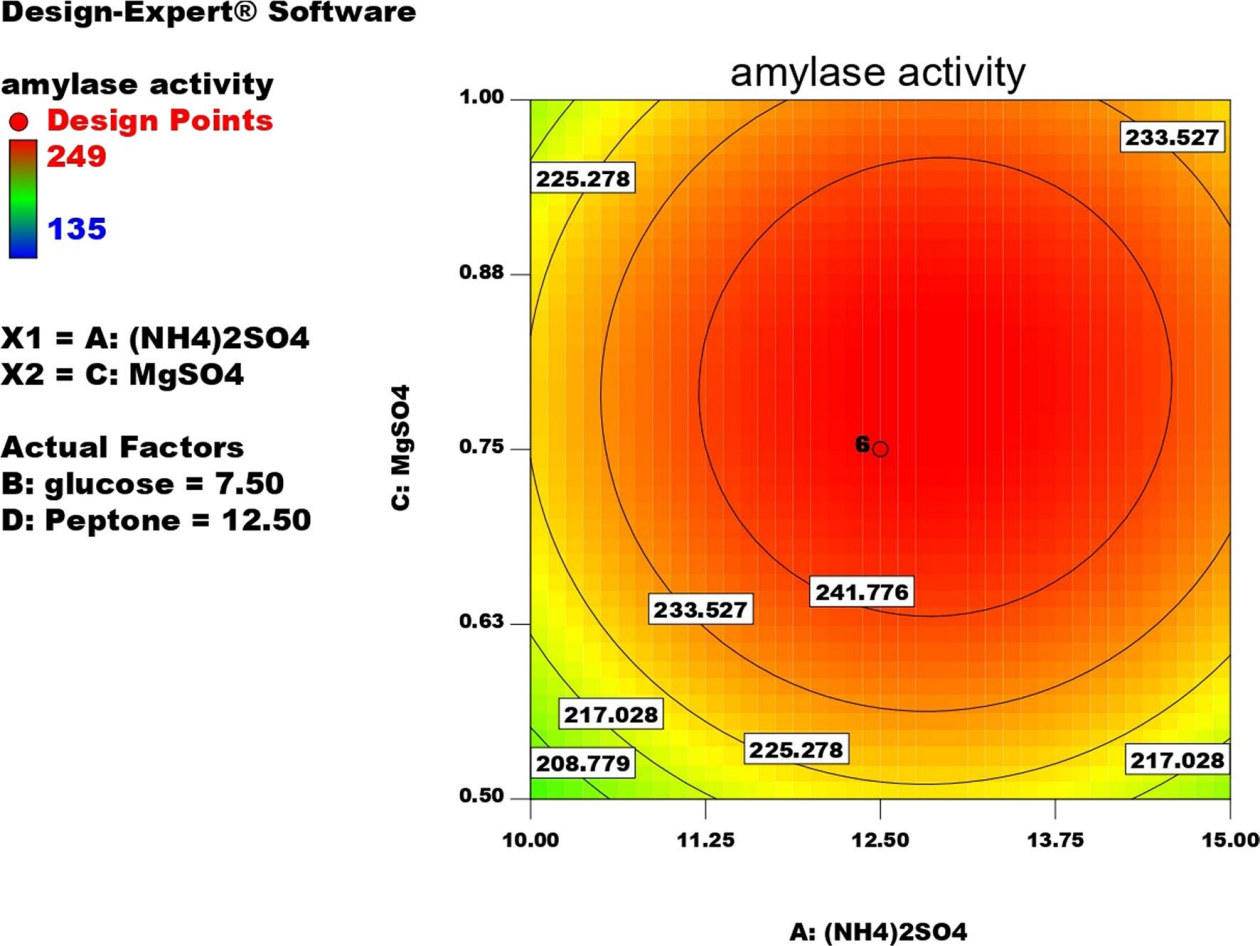
Contour plot shows interaction between (NH4)_2_SO4 Vs MgSO4

**Fig. 15:**
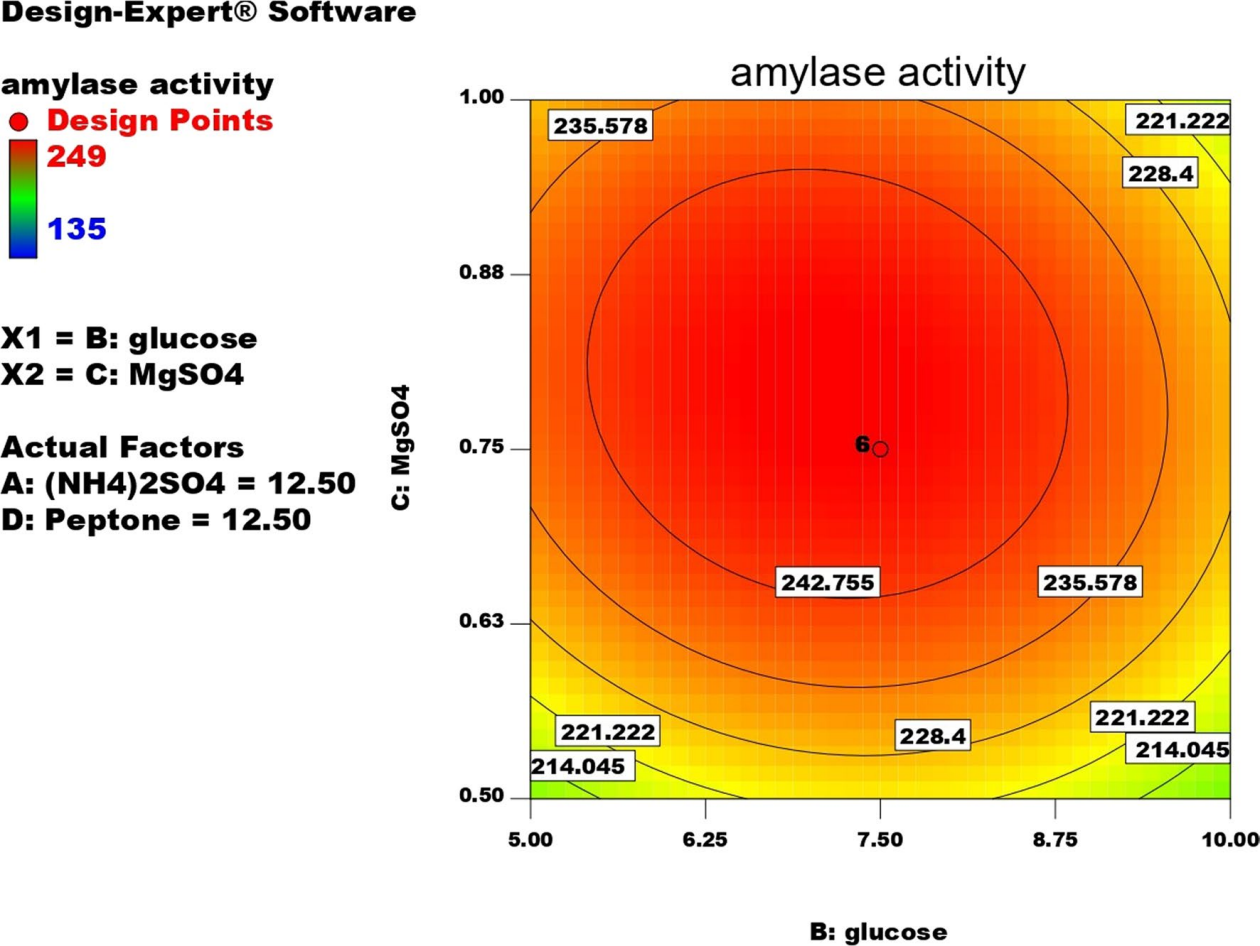
Contour plot shows interaction between MgSO4 Vs glucose

**Fig. 16:**
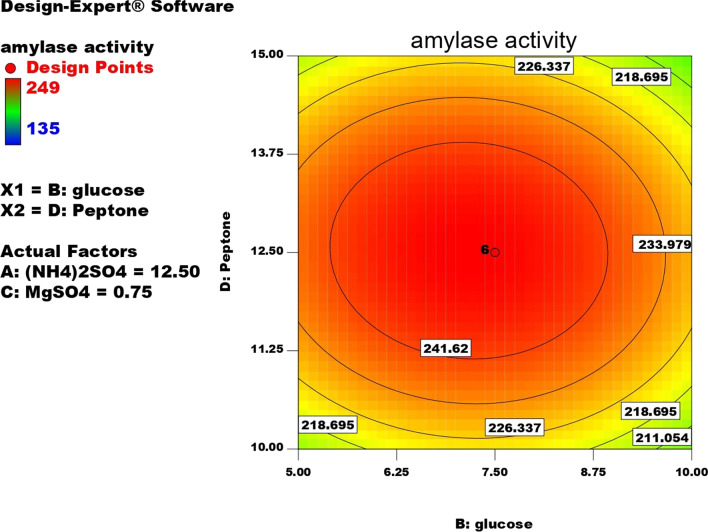
Contour plot shows interaction between glucose Vs peptone

**Fig. 17:**
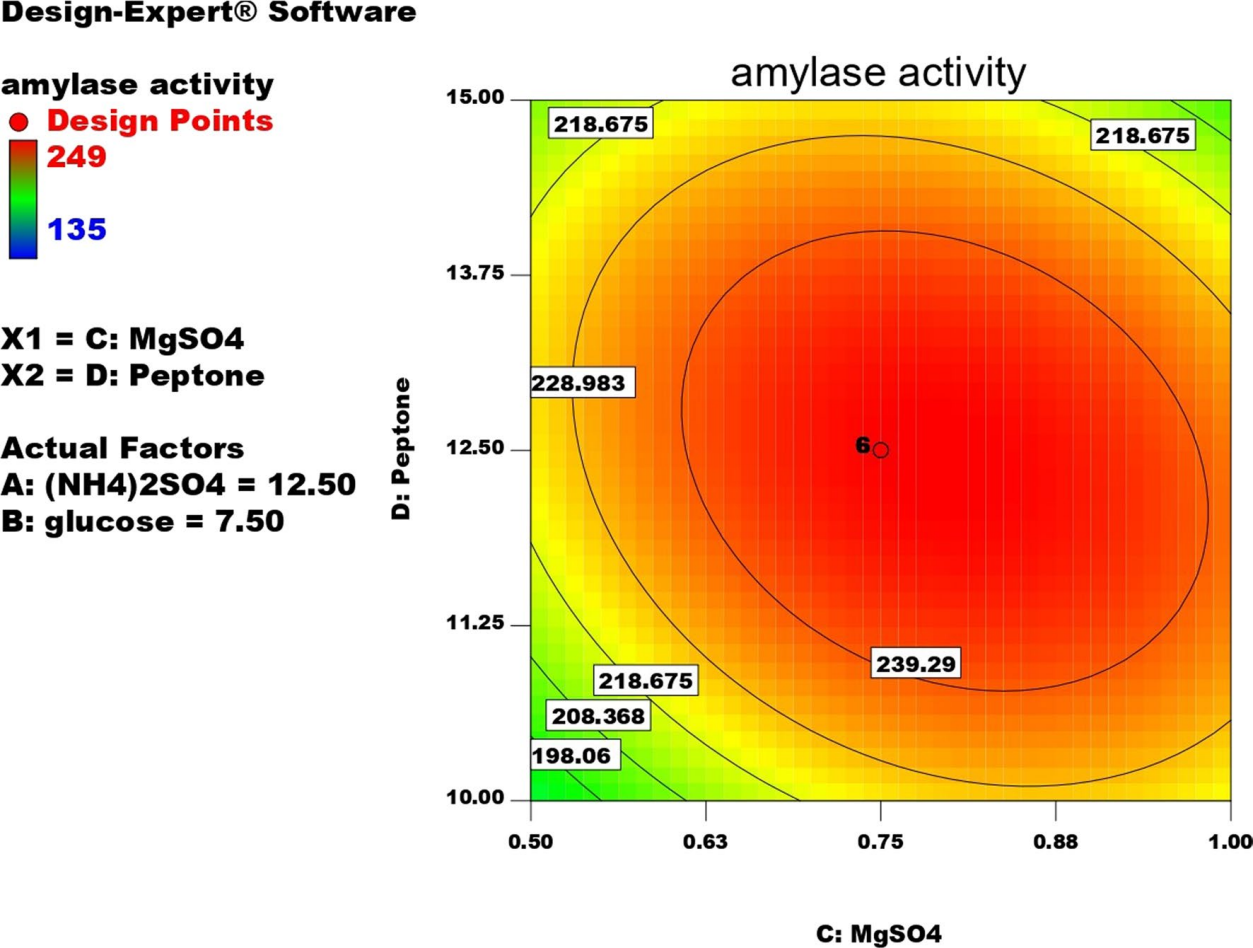
Contour plot shows interaction between (NH4)_2_SO4 and peptone

Contour plots for α-amylase production were created using two independent variables while keeping the value of the third variable constant at its central value to achieve the best conditions for maximum α-amylase production. These plots were represented by different colors, indicating different levels of α-amylase production between two independent parameters while holding the third constant. According to these graphs, each parameter had a significant impact on α-amylase production. In order to assess the precise correlations between the response and experimental levels of each variable, as well as the precise type of interaction between the variables, contour plots are widely used to represent regression equations graphically. Typically, 3D contour plots are used to analyze the optimum highest levels and interaction effects of the factors. As illustrated in Figs. 12, 13, 14, 15, 16, 17, when circular contour plots are revealed, the interaction between variables is considered negligible; however, the best interaction among variables is indicated by observation of elliptical contours, with the smallest decline in the contour representing the maximum/optimum level. The interaction effects of the variables in maximizing α-amylase yield were investigated between any 2 independent variables, while the other independent variable remained at its optimized level. Expert design software was used to analyze the results.

### Partial purification of α-amylase

#### Ammonium sulphate precipitation

Alpha-mylase of interest was separated from culture filtrate by centrifugation at 10,000 rpm for 15 min, and then the collected supernatant had been utilized as crude enzyme before selective precipitation with salts such as ammonium sulfate and organic solvents like chilled ethanol. The highest α-amylase activity was shown in fractions precipitated at 60% saturation level of ammonium sulphate and 50% saturation level of ethanol, with 33.7 U/mg and 9.7 U/mg specific activity of α-amylase, respectively. Ammonium sulphate was utilized in enzyme precipitation because it is more soluble and less expensive than other salts, remains chemically unaffected in different pH, and increases enzyme stability. The concentration of ammonium sulphate relies on the equilibrium of charges found on the surface of protein and disruption of watery layer surrounding it, which causes it to settle down, as shown in Table 6. With ethanol, there was a significant loss of α-amylase activity. Ammonium sulphate precipitation, on the other hand, produced a high yield with increased fold purification. As a result, this method was chosen for amylase fractionation [24, 37]. Furthermore, Lisio [46] reported that 80% saturation was reported to be the best percentage for α-amylase precipitation from *B. subtilis* MTCC 9447. Also, *B. methylotrophicus* P11-2 α-amylase was efficiently precipitated at 80% concentration of ammonium sulphate with purification fold of 2.3 as mentioned by Xie et al. [23]. Additionally, Fincan et al. [41], have reported 70% saturation as an optimum condition for precipitation of extracellular α-amylase form *B. subtilis.*

**Table 6:** Precipitation and dialysis of α-amylase enzyme using ammonium sulphate

Saturation (%)	Total protein mg/ml	Total activity (U/ml)	Specific activity (U/mg)	Purification fold
Crude enzyme	14.8	2218	149.8	1
0–20	1.15	17	14.7	0.098
20–40	4.9	147	30	0.203
40–60	5.1	172	33.7	0.224
60–80	0.3	3	10	0.066
80–100	0	0	0	0

#### Dialysis against phosphate buffer

The obtained ammonium sulphate precipitate was centrifuged and introduced into membrane filtration using a 10,000 MW cut-off dialysis bag overnight against 0.01 M phosphate buffer at (pH = 6.5) with three changes of phosphate buffer to obtain clear dialysate, which was then dissolved in the smallest amount of phosphate buffer and enzyme activity was determined. The specific activity was obtained using a purification fold as shown in Table 6, and the enzyme assay was done under standard conditions which described previously. Other researchers’ findings revealed that purified α-amylase from *Bacillus subtilis* had specific activity (0.06 U/mg) with a purification fold of (0.54) [24].

#### Precipitation by organic chilled ethanol solution

Enzyme precipitation steps were repeated as in previous steps in ammonium sulphate method, crude enzyme was settled down by bringing the supernatant to 50% saturation by using chilled ethanol at 4 °C, best fraction precipitate was re-dissolved in 0.01 M sodium phosphate buffer at (pH 6.5), stored at 4 °C and used for determination of α-amylase activity, as shown in Table 7**,** protein content and enzyme activity was determined under standard condition of assay [24].

**Table 7:** Precipitation and dialysis of α-amylase enzyme using ethanol as an organic solvent

Ethanol PPT	Total protein mg/ml	Total activity (U/ml)	Specific activity (U/mg)	Purification fold
Crude enzyme	14.8	2218	149.8	1
0–25	2	12	6	0.043
25–50	3.3	31	9.3	0.062
50–75	3.9	30	7.6	0.050
75–100	0.8	4	5	0.033

#### Determination of molecular weight of α-amylase by SDS-PAGE gel electrophoresis

**Fig. 18:**
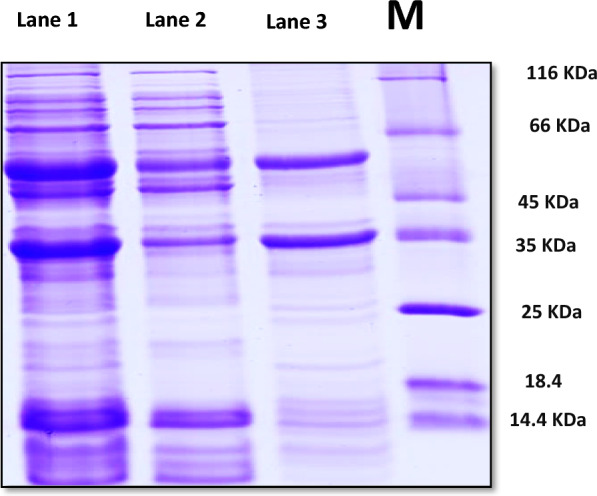
SDS-page gel electrophoresis zymogram of extracted α-amylase, Lane M: Molecular weight standard proteins, Lane 1: crude enzyme fraction, Lane 2: chilled ethanol fraction, Lane 3: Ammonium Sulphate fraction

SDS-PAGE was performed under non-reducing conditions using 7.5% stacking gel and 10% resolving gel. The protein bands were subjected to staining by Coomassie brilliant blue R-250 (Sigma Aldrich, Germany) to make them visible. Microbial amylases showed a wide range of molecular weight [47]. The molecular weight of α-amylase from *Bacillus* sp. was reported to vary between 50 and 60 kDa with only few exceptions [48]. Moreover, Liu et al. [49] found that the molecular weight of *B. licheniformis,* a thermostable α-amylase was 53.13 KDa. In the present work, the molecular weight of enzyme was specified at 58 kDa **(**Fig. 18**)** which was similar to other research findings showed that α-amylase enzyme extracted from *Geobacillus* sp. DS3 had the same molecular weight [25, 40, 50].

### Characterization of purified α-amylase

#### Thermal stability of purified α-amylase

As shown in Table 8, α-amylase activity was stable when the enzyme was stored at 4ºC for 2 h, however, it retained 95% of its activity at 35 °C for 2 h. On the other hand, increasing the temperature to 90 °C reduced the enzymatic activity by 75% because of α-amylase protein denaturation [24]. Our study supported more or less the findings of Guleria and Chatanta [51] and Sharma et al. [52] who obtained α-amylase stability at 50 °C. Also, Demirkan et al. [53] reported an α-amylase from *B. amyloliquefaciens* with 70% relative activity at 50 °C. α-amylase from *Aspergillus oryzae* S2 had been shown to exhibit maximum activity at 50 °C with a retention of 70% activity between 40 and 55 °C.

**Table 8:** Stability characters of α-amylase incubated under different temperature degrees over time (h)

Percentage of relative activity over time			
Time (h) Temp ( C)	2	4	6
4	97	95	90
35	95	91	88
55	75	70	66
65	60	55	50
75	45	40	35
90	28	22	15

#### pH stability of purified α-amylase

As shown in Table 9**,** α-amylase enzyme presented the best of its activity when kept at pH 5.5 for 2 h with retaining 95% of its activity, however increasing pH values affected the stability of α-amylase. Furthermore, changing pH of fermentation media had a significant effect on the enzymatic production due to the changes occurred in the charges of amino acids in protein molecules of α-amylase enzyme [24]. Additionally, Hmidet et al. [54] reported α-amylase from *B. licheniformis* NH1 which was highly active in the wide pH range of 5–10, with maximum activity at pH 9.

**Table 9:** Stability characters of α-amylase stored under different pH values over time

Percentage (%) of relative activity over time			
Time (h) pH	2	4	6
4.5	65	60	58
5.5	95	92	90
6.5	88	85	80
7.5	78	71	68
8.5	60	55	45
9.5	35	30	20

#### Storage stability of the purified α-amylase enzyme

As shown in Table 10, α-amylase retains 93% of its activity after storage period of 21 days, activity decreased to 70% after 40 days of storage, other researches revealed that amylase residual activity will decrease to 75% after 42 days of storage [24]. Kiran and Chandra et. al [55] studied shelf life of α-amylase from *Bacillus* sp. TSCVKK for 2 months and found it stable at 4 °C, however a loss of 15% activity was observed after 48 h when it was incubated at 30 °C.

**Table 10:** Storage stability characters of α-amylase enzyme over time

Storage period in days	Residual activity (%)
I day	98
7 days	96
14 days	95
21 days	93
35 days	80
40 days	70

### Determination of MBIC and MBEC of the purified α-amylase

Results shown in Table 11 revealed that the MBIC of commercial α-amylase against *P. aeruginosa* biofilm was 50 μg/ml and for the purified enzyme was 125 μg/ml. Moreover, the commercial enzyme recorded a MBEC value of 100 μg/ml, however the purified α-amylase enzyme showed MBEC of 250 μg/ml. This effect could be explained by the study of Lahiri et al. [56] who reported that α-amylase acts on the exopolysaccharide (EPS) through the enzymatic degradation. Exopolysaccharide is considered a major component forming the biofilm matrix and so, adhesion of bacterial cells could be inhibited. Hence, this enzyme is considered a potential antimicrobial agent.

**Table 11:** MBIC and MBEC of the purified and commercial alpha-amylases

Antibiofilm evaluation tests	Purified *B. cereus* α-amylase (µg/ml)	Purified *B. cereus* α-amylase (µg/ml)
MBIC	125	125
MBEC	250	250

### Biofilm formation and inhibition assays by spectrophotometric microtiter plate method for quantitative determination of biofilm inhibition percentage

**Fig. 19:**
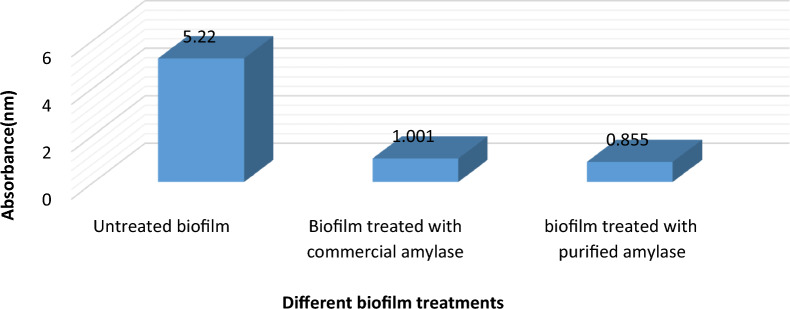
Optical density of biofilm values of *P. aeruginosa* isolate before and after treatment with commercial and purified α-amylase

As shown in Fig. 19, *P. aeruginosa* biofilm in control sample showed an absorbance at 610 nm equal to 5.22 and after treatment with commercial enzyme, the absorbance falls to 1.001 Furthermore, after treatment with the purified enzyme, absorbance fell to 0.855. As a result, the percentage of biofilm inhibition after treatment with commercial α-amylase was 80% and after treatment with the purified enzyme from *B. cereus* isolate was approximately 84%. Similar studies investigated the effect of α-amylase enzyme from *Bacillus* species on bacterial biofilms and they mentioned comparable results [25, 56, 57].

### Quantitative determination of biofilm inhibition by CLSM (biofilm thickness inhibition and live/dead cells percentage calculations)

**Fig. 20:**
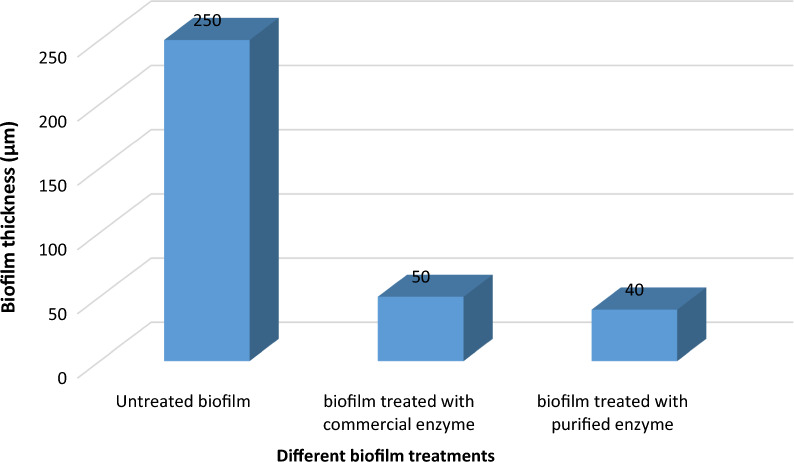
*P. aeruginosa* biofilm thickness before and after treatment with commercial and purified amylase

**Fig. 21:**
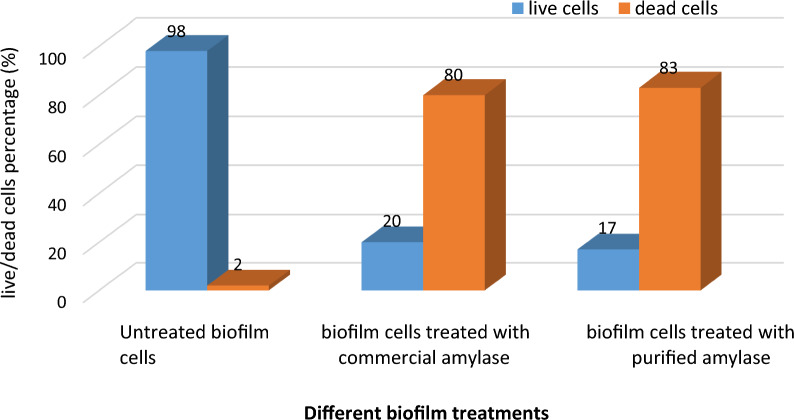
Live/dead cells percentage before and after treatment of *P. aeruginosa* biofilm samples in chamber slides with commercial and purified amylase

**Fig. 22:**
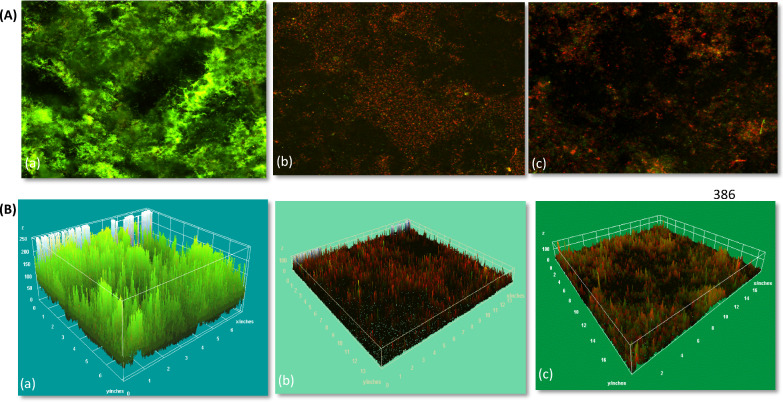
*P. aeruginosa* biofilm stained with acridine orange/propidium iodide florescent dyes before and after treatment with either commercial or purified α-amylase enzyme. (A) CLSM images of **a** untreated control biofilm showing that 98% of the biofilm was live appeared as green due to acridine orange dye that stain viable bacterial cells, **b** purified amylase-treated biofilm presenting 83% of the biofilm was red due to dead cells, and (**c**) following treatment with commercial α-amylase enzyme where 80% of the biofilm was red. (B) 3D images of control and treated biofilm showing **a** biofilm thickness of the test strain measuring 250 um while dramatically reduced biofilms were those treated with **b** purified α-amylase and (**c**) commercial α-amylase. The 3D images were analyzed by Image j software

As shown in Table 12 and Figs. 20, 21, 22, results revealed that there was approximately 84% inhibition in biofilm thickness of bacterial samples treated with the purified α-amylase enzyme recording 83% dead cells and 17% live cells, and samples treated with commercial α-amylase showed 80% inhibition in biofilm thickness presenting 80% dead cells and 20% live cells. The experiment was done using two different concentrations; 150 μg/ml and 300 μg/ml (slightly above their MBEC values) of commercial and purified α-amylase enzymes, respectively [25, 32]. It is worth mentioning that the results shown in Figs. 19, 20, 21 indicated that commercial and purified α-amylases exhibited significant effect on *P. aeruginosa* biofilm denoting great antibiofilm activity [32]. Lequette et al. [58] and Wiatr [59] had investigated a variety of enzymes including proteases, papains, α-amylase, and cellulase and concluded that the antibiofilm effectiveness of hydrolases such as amylases could be assigned to the hydrolysis of a substrate involved in bacterial adhesion like EPS and these hydrolases were effective in digesting slime layers produced by cultures of pure and mixed strains of bacteria. The outcome of the present study also parallels with this finding. Additionally, this observation was similar to the work performed by Craigen et al. [60] who also demonstrated that α-amylases possess the ability to degrade the EPS resulting in the dispersion of the cells.

**Table 12:** Biofilm thickness and live/dead cells percentages of *P. aeruginosa* biofilm before and after treatment with commercial *B. amyloliquefaciens* α-amylase and purified *B. cereus* α-amylase

Test organism	Biofilm thickness (µm) following treatment with			Percentage of Live/dead cells following treatment with		
P. *aeruginos*a	Untreated control	Commercial α-amylase	Purified α-amylase	Untreated control	Commercial α-amylase	Purified α-amylase
	250	50	40	98% live cells 2% dead cells	80% dead cells 20% live cells	83% dead cells 17% live cells

## Conclusion

Our results indicated that, from the economic point of view, *B. cereus* has a very good ability to produce extracellular α-amylase. In this work, a new amylase-producing *B. cereus* species was isolated from soil and identified at both phenotypic as well as genotypic levels then it was subjected to production process by submerged fermentation at small scale. Production parameters was optimized statistically by Plakett-Burman design and RSM CCD, then, it was found that best carbon source was starch (5 g/l), nitrogen source was peptone (12.5 g/l), metal ions was (NH4)_2_SO4 (12.5 g/l) and MgSO4 (0.75 g/l), best pH was 5.5, temperature was 35 °C and best incubation time was 48 h. Interestingly, promising similarities was found between predicted and experimental results as well as the amount of glucose, peptone, ammonium sulfate and magnesium sulfate which had a significant effect on the submerged fermentation of α-amylase enzyme. Moreover, PBD optimizes the enzymatic production by 1.5-fold and RSM optimizes the production by twofold compared to the basal medium, analysis of variance delivers high value of R-square and adjusted R-square at significant level (p ≤ 0.0075 and 0.001). The enzyme was partially purified, characterized and molecular weight was determined by SDS-PAGE electrophoresis and it was ~ 58 kDa. Antibiofilm activity was evaluated against *P. aeruginosa* biofilm spectrophotometrically. Data showed 84% antibiofilm activity and CLSM recorded 83% reduction in the biofilm thickness with 17%/83% live/dead cells percentage. Hence, our study indicated that *B. cereus* α-amylase was a good candidate as antibiofilm and could be used for cost-effective clinical applications. Thus, regardless of the concentration, purified α-amylase enzyme had the same antibiofilm activity as commercial enzyme. As a result, this study provides compelling evidence that a hydrolase bacterial enzyme such as α-amylase could be a useful biofilm inhibiting agent in clinical applications.

## Data Availability

All datasets generated for this study are included in the manuscript.
